# Escape from Autologous Neutralizing Antibodies in Acute/Early Subtype C HIV-1 Infection Requires Multiple Pathways

**DOI:** 10.1371/journal.ppat.1000594

**Published:** 2009-09-18

**Authors:** Rong Rong, Bing Li, Rebecca M. Lynch, Richard E. Haaland, Megan K. Murphy, Joseph Mulenga, Susan A. Allen, Abraham Pinter, George M. Shaw, Eric Hunter, James E. Robinson, S. Gnanakaran, Cynthia A. Derdeyn

**Affiliations:** 1 Department of Pathology and Laboratory Medicine, Emory University, Atlanta, Georgia, United States of America; 2 Emory Vaccine Center at Yerkes National Primate Research Center, Emory University, Atlanta, Georgia, United States of America; 3 Zambia Emory HIV Research Project, ZEHRP, Lusaka, Zambia; 4 Zambia Blood Transfusion Service, Lusaka, Zambia; 5 Department of Global Health, Rollins School of Public Health, Emory University, Atlanta, Georgia, United States of America; 6 Public Health Research Institute, Newark, New Jersey, United States of America; 7 New Jersey School of Medicine, University of Medicine and Dentistry, Newark, New Jersey, United States of America; 8 Department of Medicine, University of Alabama at Birmingham, Birmingham, Alabama, United States of America; 9 Department of Pediatrics, Tulane University School of Medicine, New Orleans, Louisiana, United States of America; 10 Los Alamos National Laboratory, Los Alamos, New Mexico, United States of America; National Institutes of Health-NIAID, United States of America

## Abstract

One aim for an HIV vaccine is to elicit neutralizing antibodies (Nab) that can limit replication of genetically diverse viruses and prevent establishment of a new infection. Thus, identifying the strengths and weaknesses of Nab during the early stages of natural infection could prove useful in achieving this goal. Here we demonstrate that viral escape readily occurred despite the development of high titer autologous Nab in two subjects with acute/early subtype C infection. To provide a detailed portrayal of the escape pathways, Nab resistant variants identified at multiple time points were used to create a series of envelope (Env) glycoprotein chimeras and mutants within the background of a corresponding newly transmitted Env. In one subject, Nab escape was driven predominantly by changes in the region of gp120 that extends from the beginning of the V3 domain to the end of the V5 domain (V3V5). However, Nab escape pathways in this subject oscillated and at times required cooperation between V1V2 and the gp41 ectodomain. In the second subject, escape was driven by changes in V1V2. This V1V2-dependent escape pathway was retained over time, and its utility was reflected in the virus's ability to escape from two distinct monoclonal antibodies (Mabs) derived from this same patient via introduction of a single potential N-linked glycosylation site in V2. Spatial representation of the sequence changes in gp120 suggested that selective pressure acted upon the same regions of Env in these two subjects, even though the Env domains that drove escape were different. Together the findings argue that a single mutational pathway is not sufficient to confer escape in early subtype C HIV-1 infection, and support a model in which multiple strategies, including potential glycan shifts, direct alteration of an epitope sequence, and cooperative Env domain conformational masking, are used to evade neutralization.

## Author Summary

A significant obstacle to developing an HIV vaccine is the potential for the virus to escape from the immune response induced by immunization. We previously showed that subjects in a Zambian cohort developed potent neutralizing antibody responses shortly after becoming infected by subtype C HIV-1, and here we have extended those findings to demonstrate that cycles of viral escape occurred in two of these subjects despite a potent immune response. We investigated the determinants of immune escape, and found that a single common mutational pathway was not sufficient to facilitate viral escape. Instead, we demonstrate that multiple strategies, including potential changes in glycosylation pattern, direct alteration of an epitope sequence, and cooperative envelope interactions, were used independently or together to evade neutralization. We also recovered individual monoclonal antibodies from one of the subjects and found that a single mutation can confer escape from different neutralizing antibody specificities. The studies demonstrate the remarkable flexibility of subtype C HIV-1, and suggest that the envelope glycoproteins are uniquely equipped to adjust to the specific properties of the immune response in each newly infected host.

## Introduction

The current AIDS pandemic is the result of genetically diverse viral subtypes and circulating recombinant forms (CRFs) of HIV-1 group M, of which subtypes A, C, and D account for a majority of infections worldwide [1],[2]. A key source of this genetic diversity is the viral *env* gene, which encodes the envelope (Env) glycoproteins, gp120 and gp41 (reviewed in [3]). On the virion, monomers of non-covalently associated gp120 and gp41 subunits trimerize to form ‘spikes’, and together these facilitate entry into a target cell. Env has a complex conformation and undergoes substantial rearrangements in both subunits upon gp120 binding to CD4 and coreceptor [4],[5],[6]. Env also contains the principal targets for neutralizing antibodies (Nab) [7],[8], and epitopes are targeted in both Env subunits [9]. However, many potential neutralization targets are transiently or not exposed on the trimeric form of virion-associated Env, including the V3 domain, CD4-induced epitopes, and the CD4 binding site [10],[11],[12]. Despite these limitations, most HIV-1 infected patients develop robust Nab responses against their autologous virus, particularly those infected with subtype C [13],[14],[15],[16],[17],[18],[19].

To confer potent and broad neutralization, it is expected that an epitope will need to possess at least four properties: (i) exposure on the virion-associated native Env trimer, (ii) conservation across diverse HIV-1 variants, (iii) immunogenicity, and (iv) lack of autoreactivity. To date, there are no epitopes that meet these criteria. However, our knowledge of the epitopes that are recognized by Nab during natural infection with diverse HIV-1 is somewhat limited. It is not known which or how many epitopes are targeted by the initial autologous Nab response, what proportion of these epitopes is strain-specific or shared, how antigenicity differs between patients or viral subtypes, or what the predominant escape mechanisms are. Furthermore, there is mounting evidence that at least some of these parameters differ between HIV-1 subtypes (reviewed in [20]). We, and others, have characterized the autologous Nab response as it first develops, and have observed high titers of autologous Nab activity against the infecting strain in most (but not all) patients [13],[14],[15],[16],[17],[18],[19]. In addition we have reported higher Nab titers in subtype C infected patients compared to subtype B infected patients that were evaluated in parallel, prompting our group and others to propose that there are differences in antigenicity between subtype B and C Envs [15],[20],[21],[22]. Recent studies honed in on regions that could be involved in early subtype C autologous Nab responses, and these included the V1V2 hyper-variable domain and the C3 to V4 sub-region of gp120 [23]. However, these regions could not account for all of the Nab activity present in patient plasma, suggesting the involvement of additional determinants. Furthermore, we have demonstrated that both V1V2-dependent and -independent pathways are utilized for escape from Nab during chronic subtype C infection [24]. In addition, we have shown a strong association between mutations in the α2 helix region of C3 and neutralization resistance, although these mutations did not directly alter neutralization sensitivity when transferred between sensitive and resistant Envs from linked transmission partners [25].

Temporal studies of HIV-1 have demonstrated that HIV-1 undergoes recurrent cycles of escape from autologous Nab [17],[19],[26],[27], and escape also occurred during infection with a chimeric SIV-HIV-1 (SHIV) virus in response to vaccine-induced Nab [28]. Yet, our knowledge of the specific molecular events that lead to escape remains incomplete. Shifting carbohydrate moieties in and around the outer surface of gp120, as well as changes in the hyper-variable domains, have been proposed as general mechanisms used by HIV-1 and SHIV to alter neutralization epitopes, although most of these studies are based on subtype B Envs. Nab escape in SHIV-infected macaques has been shown to involve glycan changes in the V1, V2, and V3 domains, perhaps by shielding conserved epitopes such as the CD4 binding site [29],[30],[31]. Consistent with this finding, a recent study identified V1V2 as the major determinant of strain-specific autologous Nab in macaques infected with two different strains of SHIV [32], and this domain was also shown to be the principal determinant of inherent Nab resistance for HIV-1 strain JRFL [10]. Subtype B HIV-1 can also escape from autologous Nab by shifting N-linked carbohydrates on the outer domain of gp120 with little involvement of V1V2 [17]. Others have demonstrated that subtype B viral escape could also occur in the absence of frank changes in glycosylation, with no clear mutational pattern emerging [33]. In experimental SIVmac infection, the emergence of N- and O-linked carbohydrates in the V1 and V4 hyper-variable domains has been shown to confer escape from autologous neutralization [34],[35],[36]. Furthermore, the presence of specific glycans in V1 reduced the immunogenicity of SIVmac in the context of an experimental infection [37]. Taken together, these studies hint at the complexity of HIV-1 neutralization and escape, but also suggest that common themes may exist.

Thus, unlike cytotoxic T lymphocyte (CTL) epitopes and their escape mutations, which are frequently predicted by the association of viral sequence polymorphism and HLA alleles, Nab epitopes and escape pathways in Env can be inherently difficult to identify based on sequence alone. We have therefore undertaken a molecular approach to define these in subtype C HIV-1 Env during early infection. Using a pseudovirus-based assay that facilitates evaluation of individual, patient-derived Envs, we analyzed the neutralizing ability of longitudinal plasma samples against contemporaneously-derived, autologous Envs from two subtype C seroconvertors who generated potent Nab against the infecting Env. Sequential neutralization escape variants emerged in both patients, and we used Env domain exchange and site-directed mutagenesis approaches to map the pathways involved in Nab escape at multiple time points throughout the first two years of infection. Nab escape in these two patients was driven by V1V2-dependent and -independent pathways, and these pathways exhibited different levels of stability over time. Yet, spatial representation of the sequence changes in gp120 indicated that immune pressure was directed at the same Env regions in both subjects. The derivation of autologous monoclonal antibodies (Mabs) from one patient demonstrated how a single potential glycan change in V1V2 afforded simultaneous resistance against multiple antibodies. These studies therefore provide a detailed look at Nab escape in subjects recently infected with the most predominant subtype worldwide, and demonstrate that the flexibility of Env facilitates the use of multiple mechanisms.

## Results

### Continuous cycles of neutralization escape occur throughout early subtype C infection

**Figure 1:**
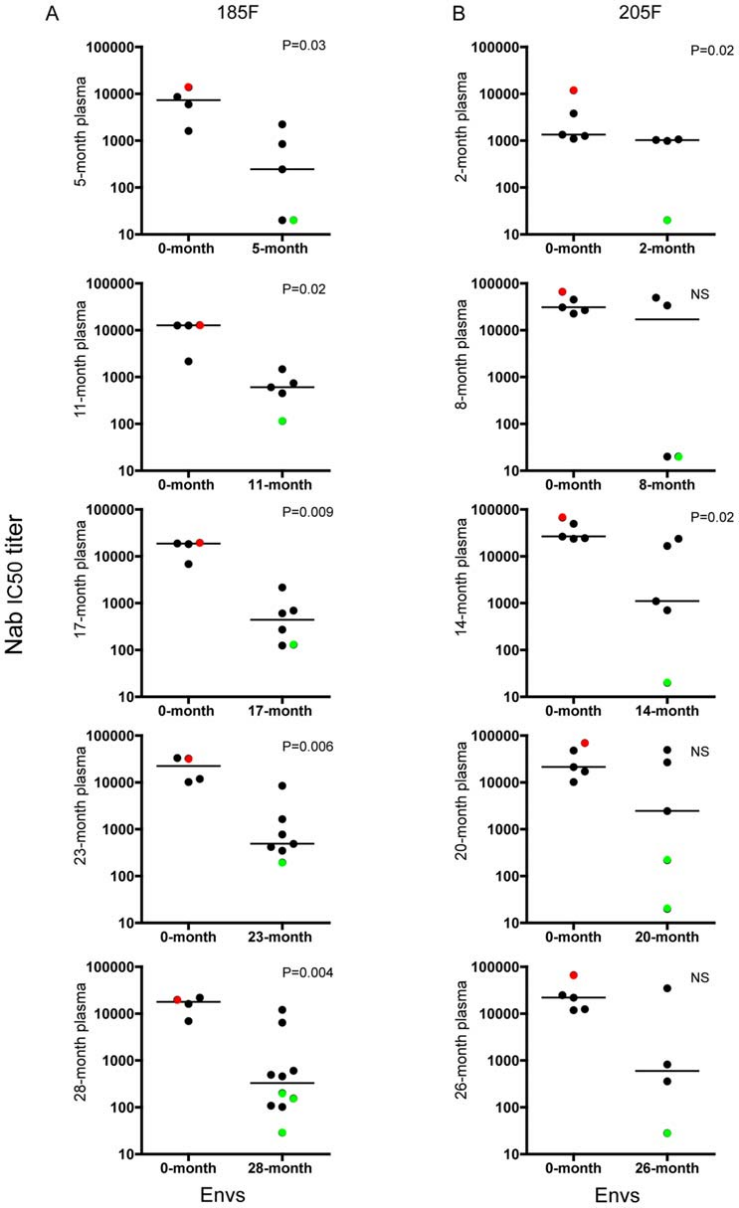
Cycles of escape from autologous Nab in 185F and 205F. Each panel represents a longitudinal plasma sample where Nab activity was evaluated for (A) subject 185F (5, 11, 17, 23, and 28 months) or (B) subject 205F (2, 8, 14, 20, and 26 months). Each data point represents the Nab IC50 titer (shown on the vertical axis on a log10 scale) for a single plasma-Env combination calculated from the virus infectivity curve using the Excel growth function. The Nab IC50 titers against the 0-month Envs and the contemporaneous Envs are shown for each longitudinal plasma sample (labeled along the vertical axis), and the horizontal bars indicate the median for each group of Envs. A Mann-whitney analysis was used to test for statistically significant differences between the Nab sensitivity of the 0-month and contemporaneous Envs, and p-values less than 0.05 were considered significant. Red dots indicate the Nab IC50 titer for 0-month Env that was used as a background for the chimeras. Green dots indicate the Nab resistant Envs from each time point that were selected and used to generate the chimeras.

We previously demonstrated that 9 out of 11 subtype C infected subjects from the ZEHRP cohort developed robust autologous Nab responses against the infecting Envs, with 50% inhibitory (IC50) titers often exceeding 1∶3,000 within the first few months of infection [15]. For two of the subjects who developed potent autologous Nab and were identified as viral p24 antigen positive (Table 1), we sampled the emerging quasispecies by single genome PCR amplification, cloning, and sequencing of biologically functional *env* genes from longitudinal plasma and PBMC DNA samples. For both subjects, the 0-month Envs were cloned at the first seropositive time point estimated to be within 48 days of infection, and longitudinal timing was calculated in months from this point forward [38]. Samples from five subsequent time points over the first two years of infection were evaluated. A subset of Envs was chosen to represent the diversity of the circulating quasispecies at each time point (see arrows in Fig. S1A and B), and was evaluated for sensitivity to neutralization by each contemporaneous (simultaneously collected) plasma sample using the JC53-BL (Tzm-bl) pseudovirus assay [15],[24],[25]. The IC50 Nab titer for each plasma-Env combination was calculated from each virus infectivity curve using a growth function. Fig. 1 shows that the median IC50 titer of the 0-month Envs (designated according to the first seropositive time point; see Table 1) was higher than the contemporaneous Envs at each time point, indicating repeating cycles of neutralization resistance. This difference in median IC50 titer was statistically significant at all time points for 185F. However, for 205F, the median IC50 titer differed significantly at only two time points, probably due to the wide range of Nab sensitivities observed for the contemporaneous Envs of this subject (Fig. 1B). Nevertheless, Nab-resistant variants were present at each time point and these were neutralized by subsequent plasma samples, indicating continued induction of a de novo Nab response (data not shown).

**Table 1:** Seroconvertors from ZEHRP that were evaluated for Nab escape.

Subject	Viral load^1^	Viral load^2^	Visit month	Last seroneg date	First seropos date	p24 pos date^a^	First sample date	Estimated days from infection^b^
185F	54,377	91,678	72	6-Aug-02	17-Aug-02	6-Aug-02	17-Aug-02	33
205F	6,125	400	18	1-Mar-03	27-Mar-03	1-Mar-03	27-Mar-03	48

Viral load: RNA copies/ml in plasma at the first sample date^1^ and 11months later^2^.

Visit month: time period between enrollment into ZEHRP as a discordant couple and the first seropositive visit.

a 185F was also p24 doubtful on 17-Aug-02.

b 22 days prior to the Ag+ Ab- date for 185F and 205F as described in [38].

### Plasma Nab in subjects 185F and 205F possesses moderate breadth against heterologous Envs

**Figure 2:**
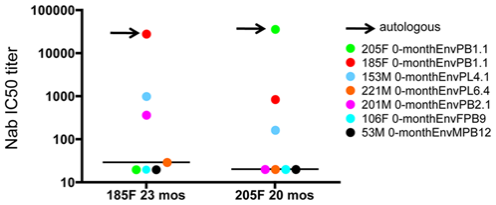
Moderate neutralization breadth of plasma from 185F and 205F against heterologous 0-month Envs. A single plasma sample from 185F (23-months) and 205F (20-months) was evaluated for neutralizing activity against heterologous Envs acquired during acute/early subtype C infection of six subjects. The Nab IC50 titer for each plasma-Env combination is shown on the vertical axis on a log10 scale. Each data point represents a single plasma-Env combination. Arrows indicate the Nab IC50 titer for the autologous plasma-Env combination; all other data points represent heterologous Envs. The plasma sample is indicated below each point plot.

To gauge whether breadth developed within the window of evaluation, cross-neutralizing activity of a single plasma sample from subject 185F (23-months) and subject 205F (20-months) was measured against heterologous 0-month subtype C Envs from six other subjects in the same cohort (Fig. 2). Consistent with previous studies of early subtype C infection [15],[16], Nab in 185F and 205F was mostly strain-specific. However, each of these plasma samples neutralized 2 of the 6 heterologous Envs with an IC50 of greater than 1∶100, and in two cases approaching 1∶1000. Interestingly, 205F plasma potently neutralized the 185F Env (red dot on right point plot), but the reciprocal was not observed (green dot on left point plot). Thus, Nab in these plasma samples was directed against predominantly strain-specific targets, but were also capable of recognizing some common epitopes by approximately two years after infection.

### Selection of Nab resistant ‘escape’ variants for further study

For 185F, the median Nab IC50 titer for the 0-month Envs was significantly greater than the contemporaneous Envs at every time point using a Mann-whitney test (Fig. 1A). Using the criteria of at least a 100-fold decrease in sensitivity to neutralization compared to the median of the 0-month Envs, a resistant Env (highlighted in green) from each time point was selected for in-depth investigations into Nab escape. At 28-months, three different Nab resistant Envs were selected because the phylogenetic tree indicated that multiple lineages of resistance were circulating at this time point (Fig. S1A). For 205F, the difference between 0-month and contemporaneous Envs reached significance at only two of the five time points, although there were Envs at each time point for which Nab activity was undetectable at the highest dilution of plasma tested (1∶20, Fig. 1B). These Envs, which were often 1000-fold less sensitive to Nab than the median of the 0-month Envs, were selected for detailed studies of Nab escape. Two genetically diverse Envs from distinct lineages were also selected to represent the 20-month time point in 205F (Fig. S1B).

### Early Nab escape involves different escape pathways in subjects 185F and 205F

We next investigated the adaptations that were responsible for escape from contemporaneous Nab in 185F and 205F. The 0-month Envs were potently neutralized by plasma from all subsequent time points (Fig. 1A and B) and were used to provide a neutralization sensitive background, which remained more than 95% conserved at the amino acid level with subsequent variants and could be used to investigate the molecular determinants of escape for each Nab resistant variant. To do this, two approaches were used: (i) where sequence changes were limited in the Nab resistant Env, site-directed mutagenesis was used to introduce potential escape mutations into the 0-month Env and (ii) where multiple sequence changes were present in the Nab resistant Env, larger Env subregions (i.e. V1 to V5, V3 to V5, V1V2, etc.) were transferred from the Nab resistant Env into the 0-month Env. The neutralization sensitivity of the chimeric and parental Envs was then evaluated using plasma contemporaneous with the Nab resistant Env.

**Figure 3:**
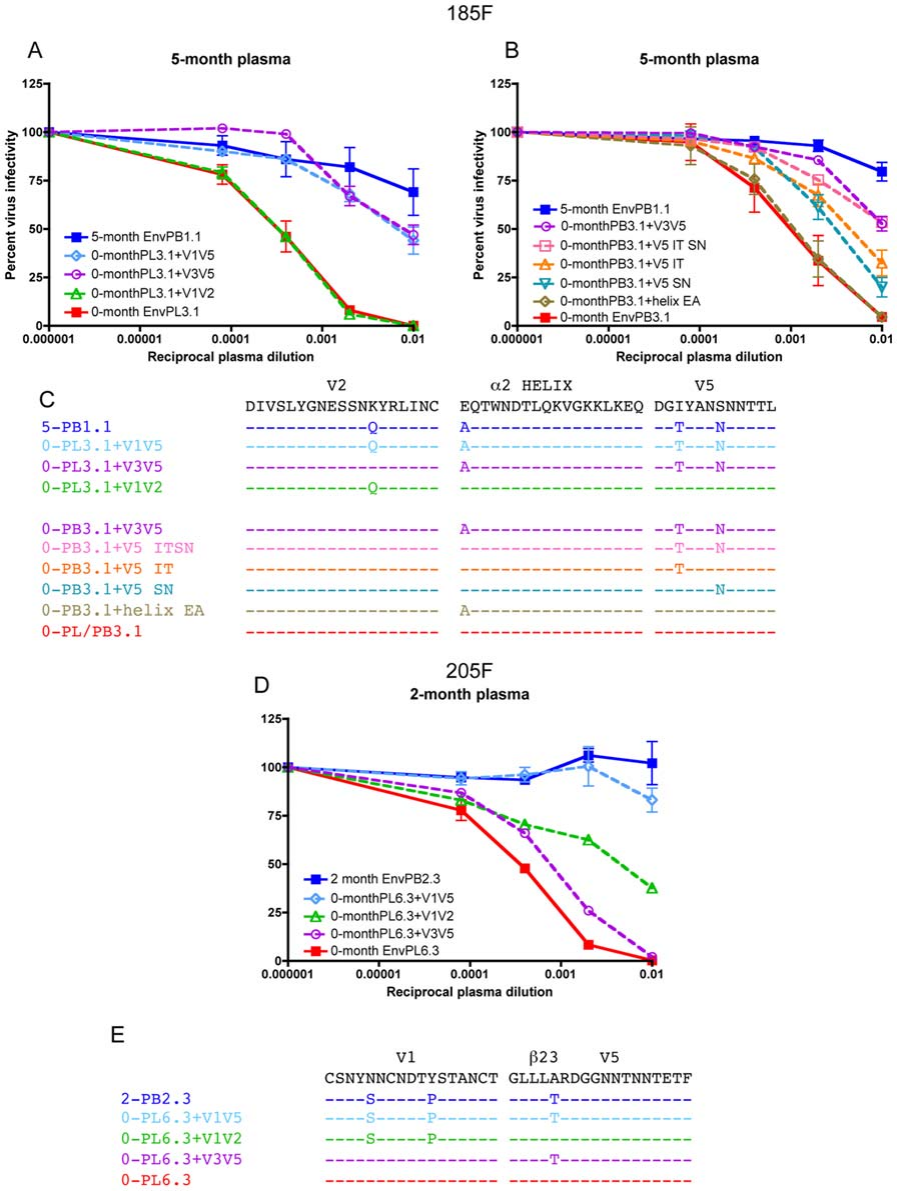
Early escape from neutralization in 185F and 205F requires different pathways. Neutralization of escape variants 185F 5-month PB1.1 (A and B), and 205F 2-month PB2.3 (D) and chimeras or mutants from each of these Envs in the corresponding 0-month Env was evaluated using contemporaneous plasma. Pseudoviruses were created by expressing each Env with an HIV-1 *env*-deficient backbone, and their infectivity for JC53-BL13 (Tzm-bl) cells was evaluated in the absence or presence of serially-diluted patient plasma with luciferase as a quantitative measure. Percent virus infectivity relative to no test plasma is plotted on the vertical axis; the reciprocal of the plasma dilution is plotted along the horizontal axis on a log10 scale. Each curve represents one Env against serial plasma dilutions, and error bars represent the standard deviation of at least two independent experiments using duplicate wells. The legends list the parental Nab resistant Env followed by the chimeric and mutant Envs created in the 0-month Env background. Amino acid alignments are shown to indicate the sequence differences between the different Envs for 185F (C) and 205F (E). The color of the text corresponds to the curves on the graph. Only regions that contained differences are shown.

For 185F, the 5-month Nab resistant EnvPB1.1 was chosen to determine which sequence adaptations were responsible for early Nab escape. Fig. 3A demonstrates that the chimeras containing either the region spanning the V1 loop through the end of the V5 domain (V1V5) or the V3 loop through the V5 domain (V3V5) from the 5-month Env displayed a level of resistance similar to the parental Env. However, the Nab sensitivity of the chimera containing the entire V1V2 domain (V1V2) was unchanged compared to the 0-month Env, despite a K192Q change in V2 (Fig. 3C). We therefore surmised that Nab resistance was heavily dependent upon the V3V5 subregion, in which there were 3 residues that differed between 0-month EnvPB3.1 and 5-month EnvPB1.1. These changes were an E335A in the first position of the α2 helix, and two changes in the V5 region: I459T, which may also impact CD4 binding, and S463N (Fig. 3C; based on HXB2 numbering). None of these changes altered any of the predicted N-linked glycosylation sites. To assess its individual contribution to Nab resistance, each amino acid change was introduced into the 0-month EnvPB3.1. The V5 mutations I459T and S463N each independently produced a decrease in neutralization sensitivity, while these mutations combined recapitulated the Nab resistance level of the V3V5 chimera (Fig. 3B). In contrast, the E335A change in the α2 helix did not decrease neutralization sensitivity when introduced by itself into the 0-month Env (Fig. 3B). Together, these findings indicate that the combined V5 mutations facilitated neutralization resistance at 5 months, while the changes in the α2 helix and the V2 loop did not contribute to a detectable level, at least within the context of the 0-month Env.

A different scenario was observed for early escape in 205F. For the 2-month escape variant EnvPB2.3, the V1V5 region contained determinants for Nab resistance (Fig 3D), and this entire region differed from 0-month EnvPL6.3 by only 3 amino acids (Fig. 3E). Two changes were located within V1 (an N134S substitution that introduced a potential N-linked glycosylation site near the N-terminal V1V2 stem and a Y140P substitution; numbered according to Fig. S2). In contrast to 185F, introduction of the two changes in V1 decreased Nab sensitivity by more than 10-fold in the context of the 0-month Env (Fig. 3D). A third change, A453T, was located within the β23 region and has the potential to impact CD4 binding (Fig. 3E). This residue by itself reduced Nab sensitivity by about 3-fold (Fig. 3D). Thus, for 205F, complete Nab resistance at 2-months could be achieved by sequence changes in V1, including addition of a potential N-linked glycan, and a change in a region involved in CD4 contact [39].

### Nab escape in subject 185F involves more complex determinants over time

**Figure 4:**
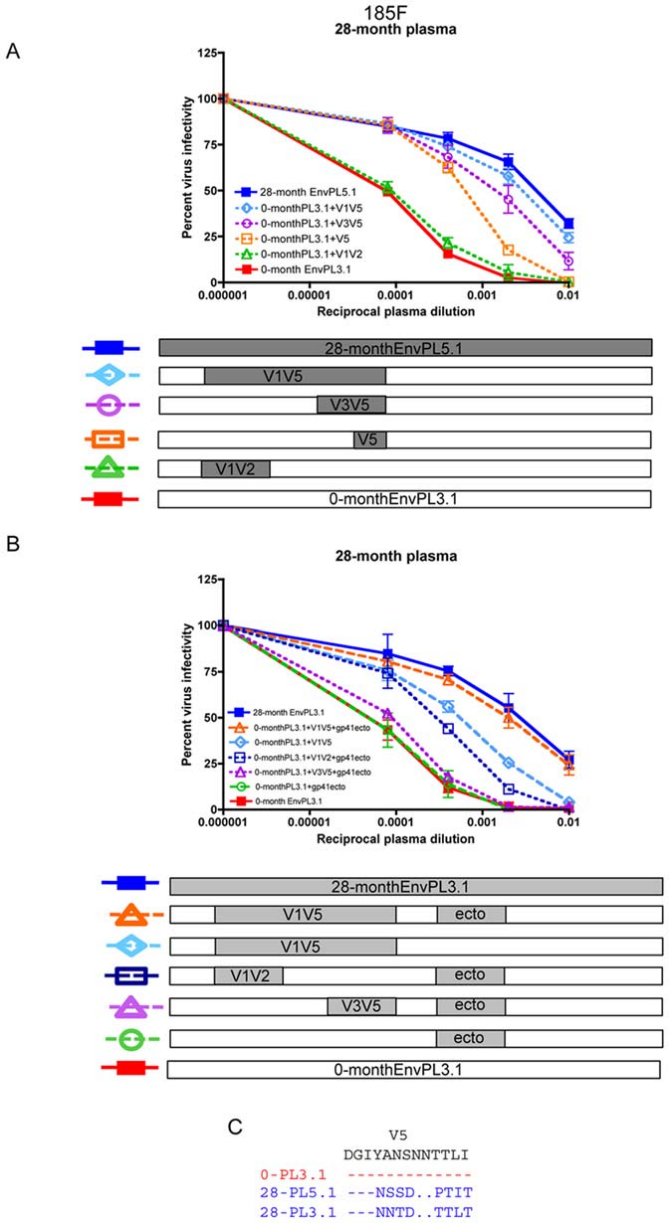
185F Nab resistant variants at 28-months utilize distinct escape pathways. Neutralization of 28-month EnvPL5.1 (A) and 28-month EnvPL3.1 (B) and the chimeric Env pseudoviruses generated from each of these Envs in the 0-month Env background was evaluated using the 28-month plasma sample in JC53-BL13 cells with luciferase as a quantitative measure. Percent virus infectivity is plotted against the reciprocal of the log10 plasma dilution. Error bars represent the standard deviation of at least two independent experiments using duplicate wells. The panels below each graph indicate the region that was transferred from the 28-month Nab resistant Env (PL5.1 in A and PL3.1 in B; gray boxes) into 0-month EnvPL3.1 (white boxes). (C) Amino acid alignment of the V5 region for the 0-month and 28-month Envs.

Although the mutagenesis studies strongly suggested that the changes in V5 at 5-months were Nab escape mutations, these specific residues were not maintained in the subsequent Nab resistant Envs (Fig. S3). However, the sequence of the V5 domain continued to evolve over time, suggesting ongoing selective pressure from Nab. At the last time point analyzed, 28-months, genetically distinct lineages of Nab escape variants were circulating (Fig. S1A). The chimera-mapping approach revealed that these different Env variants had acquired resistance through at least two distinct mutational pathways (Fig. S4, see bottom 3 panels). More detailed mapping revealed that for 28-month EnvPL5.1, the V5 domain continued to contribute to Nab resistance (Fig. 4A), retaining the major escape pathway operative at 5-months. By contrast, 28-month EnvPL3.1 achieved a similar level of resistance through an escape pathway that required the gp41 ectodomain in addition to the cognate V1V5 domain (Fig. 4B). For this Env, the V1V5 region from the 28-month Env independently conferred only partial escape onto the 0-month Env, while insertion of the 28-month ectodomain alone had no effect on Nab sensitivity. Further dissection revealed somewhat surprisingly that the V1V2 domain was a major contributor to Nab resistance in the context of the gp41 ectodomain, while the V3V5 region in this context did not appear to contribute to escape. Thus, a cooperative interaction between the gp41 ectodomain and V1V2 appeared to be conferring Nab resistance in this Env.

### Nab escape in 205F is primarily determined by V1V2 over time

Having found that changes in the V1V2 and β23 regions drove early escape from Nab in 205F, we investigated whether this pattern was maintained over time. Env chimeras were created for 205F using Nab resistant Envs from five time points over a 26-month follow-up period (Fig. 1B) and evaluated using the same approach as for 185F. V1V2 was the major determinant of Nab resistance at all time points analyzed (see Fig. S5). By contrast, the V3V5 region alone had little effect on resistance, although in combination with V1V2 it clearly contributed to escape.

**Figure 5:**
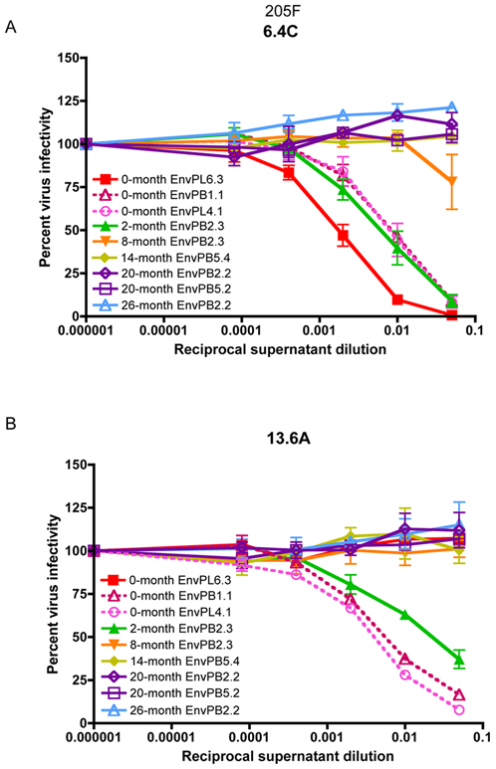
Neutralization profile of 205F Envs by autologous monoclonal antibody-containing supernatants from B cell hybridomas 6.4C and 13.6A. Neutralization activity of 6.4C (A) and 13.6A (B) B cell hybridoma supernatant was evaluated against pseudoviruses expressing autologous 205F Envs. Three different 0-months Envs are shown along with the longitudinal panel of Envs. Percent virus infectivity is plotted against the log10 of the reciprocal dilution of hybridoma culture supernatant in each graph. Error bars represent the standard deviation of at least two independent experiments using duplicate wells. 0-month EnvPB1.1 was used to screen the hybridomas for neutralization activity. B cell hybridomas from 205F were generated at 49-months post-infection.

In an effort to more precisely define Nab targets and escape pathways in 205F, B cell hybridomas were generated from viably frozen PBMC samples collected at 49 months after infection, which were the earliest available sample of this type. A 0-month Env (clone PB1.1) was used to screen for neutralizing activity in the hybridoma supernatants, and two hybridomas produced monoclonal antibodies (Mabs; 6.4C and 13.6A) that neutralized this and other 0-month Envs (Fig. 5A and B, respectively). Surprisingly, the 0-month Envs were not equally sensitive to neutralization by the two Mabs, despite being very homogeneous in sequence and potently neutralized by patient plasma (Fig. 1B). For Mab 6.4C, 0-month EnvPL6.3 was moderately more sensitive to neutralization than the other two 0-month Envs (Fig. 5A). In contrast, neutralizing activity for 13.6A was not detectable against EnvPL6.3, but the other two 0-month Envs were neutralized at levels similar to those observed with Mab 6.4C (Fig. 5B). The 2-month EnvPB2.3 was neutralized by both Mabs (Fig. 5A and B). Neutralizing activity against Envs cloned at 8-months or beyond, however, was undetectable for both Mabs (Fig. 5A and B), providing strong evidence that these Mabs could be representative of those elicited during early infection and that the later Env variants had developed resistance mutations that protected against both specificities. An identical pattern was observed for the purified 6.4C and 13.6A Mabs, with a mean IC50 against the sensitive 0-months Envs of 39 and 156 ng/ml, respectively (data not shown). Envs from 8-months and beyond were not neutralized at 10 µg/ml of either purified Mab (data not shown).

### Resistance against Mabs 13.6A and 6.4C involves loss and gain of predicted glycan addition sites in V1V2

**Figure 6:**
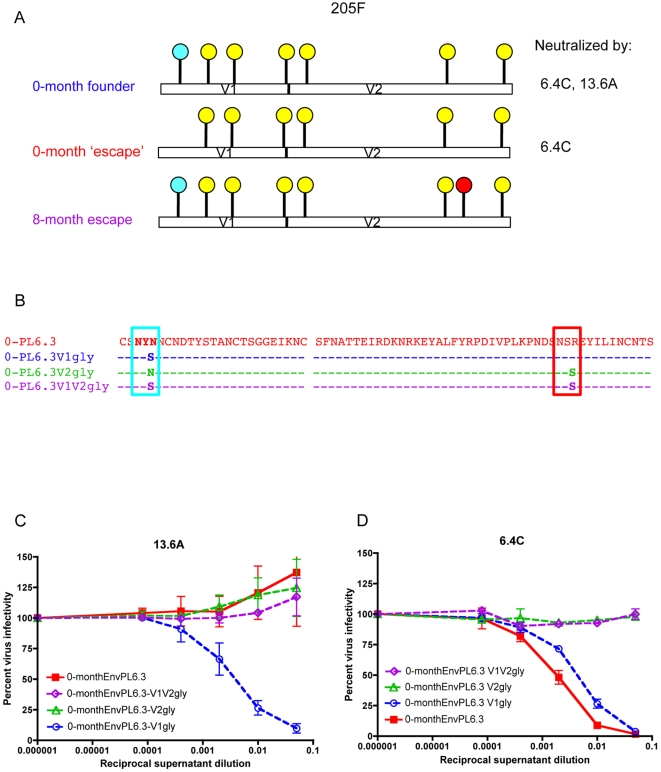
Potential N-linked glycosylation sites in V1 and V2 modulate sensitivity to Mabs. (A) Schematic diagram showing the position of potential N-linked glycans on the V1V2 sequence of the ‘founder’ 0-month Env sequence (inferred from Fig. S6), the 13.6A-resistant 0-month EnvPL6.3, and the 6.4C/13.6A-resistant 8-month EnvPB2.3. The absence of the V1 glycan shown in blue was associated with resistance against 13.6A, while addition of the V2 glycan shown in red was associated with resistance against 13.6A and 6.4C. (B) Potential N-linked glycosylation sites (NXS or NXT where X is any residue except proline) in V1V2 that were introduced singly or in combination into EnvPL6.3 are shown in blue (V1) or red (V2). The text color corresponds to the graphs in (C and D). 0-month EnvPL6.3 naturally lacks both sites. Neutralization of 0-month EnvPL6.3 and the site-directed mutants shown in panel B was evaluated for 13.6A (C) and 6.4C (D) using reduction of luciferase production in JC53-BL13 cells. Percent virus infectivity is plotted against the log10 reciprocal dilution of hybridoma supernatant. Error bars represent the standard deviation of at least two independent experiments using duplicate wells.

Neutralization of the 205F chimera panel by each Mab localized differences in sensitivity to the V1V2 domain (data not shown). Examination of the V1V2 sequences of 0- to 26-month 205F Nab resistant Envs revealed that each one differed in length, pattern of predicted glycosylation sites, and sequence (Fig. S2). However, all of the Nab resistant Envs from 8-months and beyond had acquired a mutation that created a potential glycosylation site in V2 at position 197 (highlighted in yellow in Fig. S2). Furthermore, 0-month EnvPL6.3 was the only 0-month Env that was resistant to 13.6A, and it lacked a potential N-gly site in V1 relative to the other Envs (highlighted in yellow in Fig. S2). Thus, we hypothesized that a different array of potential glycan addition sites determined the pattern of sensitivity to the two Mabs. Fig. 6A shows the naturally occurring patterns of these predicted glycan sites that were detected in the early 205F Envs with the positions of the sites of interest indicated in red (V1) and blue (V2).

To define the effects of these potential glycan addition sites in V1 and V2 on sensitivity to the two Mabs, both sites were introduced into 0-month EnvPL6.3, which carried neither (Fig. 6B). For Mab 13.6A, introduction of the V1 predicted glycan site into 0-month EnvPL6.3 resulted in a dramatic increase in neutralization sensitivity (Fig. 6C). In contrast, for 6.4C, introduction of the V1 predicted glycan produced a moderate decrease in sensitivity (Fig. 6D). Introduction of the V2 predicted glycan site into EnvPL6.3, with or without the V1 predicted glycan site, resulted in strong protection against both Mabs (Fig. 6C and D). Thus, predicted glycosylation at this site in V2 potentially tracked with protection against both Mabs (for a summary of longitudinal Envs and glycan sites see Table 2). These results also demonstrate that while both Mabs target a V1V2-dependent epitope, they recognize distinct structures.

**Table 2:** Summary of Mab neutralization data for 205F Envs.

Env	V1 glycan	V2 glycan	13.6A	6.4C
0-month PB1.1	+	−	+	+
0-month PL4.1	+	−	+	+
2-month PB2.3	+	−	+	+
0-month PL6.3 - V1gly	+	−	+	+
0-month PL6.3	−	−	−	+
0-month PL6.3 - V2 gly	−	+	−	−
0-month PL6.3 - V1V2gly	+	+	−	−
8-month PB2.3	+	+	−	−
14-month PB5.4	+shift	+	−	−
20-month PB2.2	+	+	−	−
20-month PB5.2	+	+	−	−
26-month PB2.2	+shift	+shift	−	−

The detection of a 0-month Env that was resistant to one of the Mabs was unexpected given the early timing and high sensitivity of these Envs to patient plasma. Therefore, the frequency of this predicted glycan site in V1 during acute/early infection was investigated using 21 uncloned single genome amplified V1V4 sequences from a p24-positive, antibody-negative sample (1-Mar-03) and 31 from the 0-month sample (27-Mar-03), which was antibody positive [38] (see Table 1). These sequences were combined with the five cloned 0-month Envs from this study, and a highlighter plot was created using the HIV Database (Fig. S6). Forty-four out of 57 sequences (77%) were identical throughout V1V4, and all of these contained the predicted glycan addition site in V1. Two variants (including EnvPL6.3), both from the antibody positive time point, carried an identical G to A mutation that abrogated the predicted V1 glycan addition site. Taken together, these observations provided strong evidence that the founder virus, like the 0-month Envs PB1.1 and PL4.1, carried the predicted glycan site in V1, and that the mutation in 0-month EnvPL6.3 arose shortly after transmission, but circulated only transiently.

## Discussion

### V5 mediates early escape and could serve as a Nab target

In a previous study, we demonstrated that subtype C infected seroconvertors mount robust Nab responses against their autologous viruses during the early stages of infection [15]. Here we have extended those findings to demonstrate that cycles of viral escape occur despite potent Nab and that these cycles involve multiple mechanisms and regions of Env. In subject 185F, early Nab escape required amino acid substitutions in V5 that were independent of glycosylation. It is possible that these changes directly altered an epitope in V5, as this region may be accessible to Nab on the Env trimer. However, attempts to remove Nab activity with a V5 peptide were unsuccessful, and Env chimeras in which unrelated Envs were engineered to carry the 185F 0-month V5 sequence lacked biological activity (data not shown). Thus neither of these approaches allowed definitive identification of a V5 epitope, and the latter suggested that the V5 domain itself, or the proximal region of gp120, likely evolved in concert with adjacent regions of the protein. Another possibility is that the early changes in V5 created conformational changes that protected a distinct target. The N-terminal region of V5 has been shown to contain contact sites for both CD4 and Mab b12 [39], and the escape mutations could therefore have influenced exposure of epitopes such as the CD4 binding site. These two alternatives, epitope mutation or masking, are not mutually exclusive, and it is conceivable that V5 changes could protect from more than one antibody specificity. This is clearly the case for 205F, where a single amino acid change in V2 creating a potential glycan addition site resulted in resistance against two distinct Mabs.

### Distinct escape pathways were observed within subject 185F

**Figure 7:**
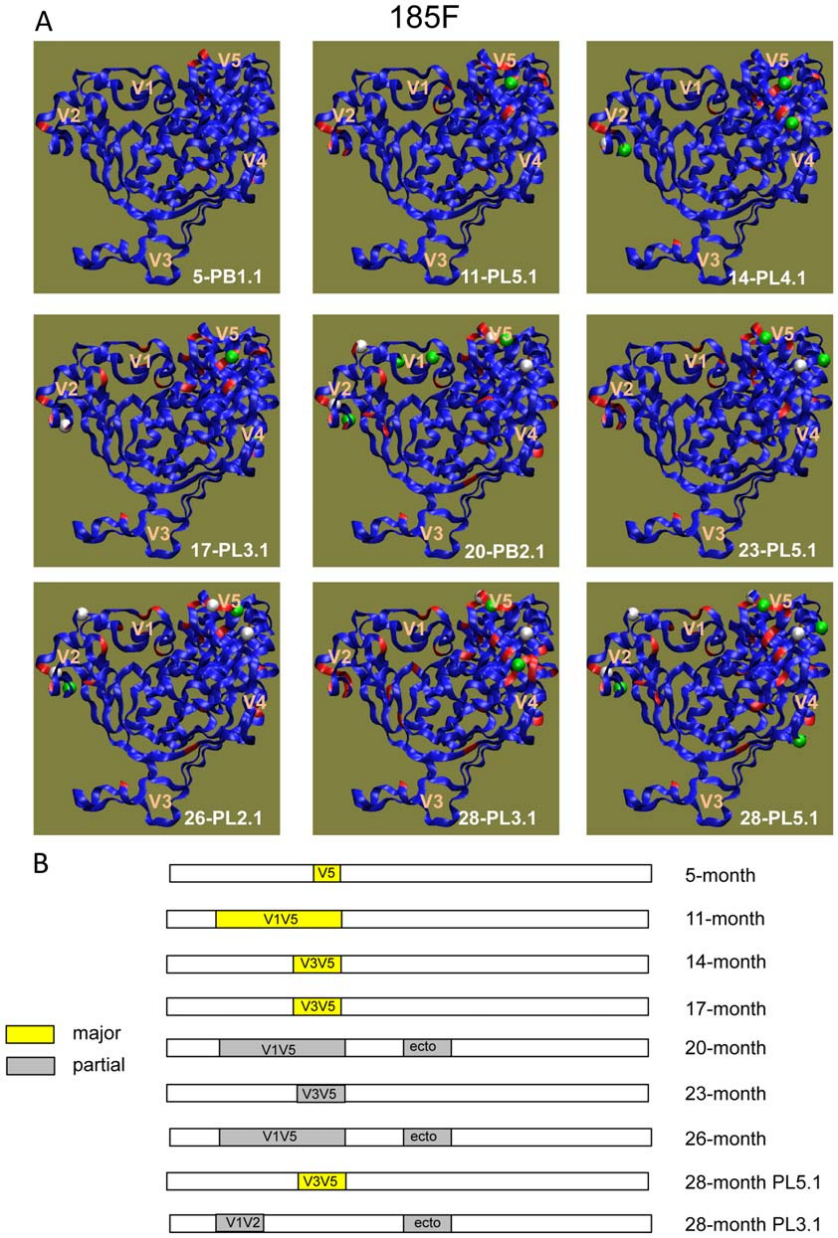
Nab escape in 185F involves multiple pathways. (A) A 3-dimensional representation of the gp120 amino acid sequence for a different longitudinal Nab escape variant Env is shown in each panel. The time point and clone is indicated in each panel. The blue gp120 backbones were generated by homology modeling of the 0-month Env sequence onto the CD4-liganded HIV-1 YU-2 gp120 structure [54], with modeled V1V2 and V3 loops as described previously [25]. Red indicates amino acid changes relative to the 0-month Env sequence. Spheres indicate changes in potential N-linked glycosylation sites (green = loss, white = gain). (B) Schematic representation of the Env domains contributing to Nab escape at each time point. Yellow indicates that a single domain had a major effect on Nab resistance; gray indicates that a particular domain contributed partially to Nab resistance. For more detail, see Fig. S4.

At later time points in subject 185F, the flexibility of the Env structure provided alternative mutational pathways to resist neutralization. Escape pathways in 185F oscillated between changes localized to the gp120 outer domain (V3V5), and conformational masking strategies that required interaction between spatially separated Env subregions (Fig. 7A and B). V5 or V3V5 was the major determinant at 5-, 14-, and 17-months, and also in one of the 28-month Nab resistant Envs (see Fig. S4 for neutralization curves). However, in Envs from two other time points (11- and 23-months), V3V5 did not independently confer resistance, but appeared to require contributions from V1V2. In Nab resistant Envs from three time points (20-, 26-, and 28-months), the gp120 V1V5 region and the gp41 ectodomain were both required for resistance. Further mapping for one of these Envs demonstrated that the V1V2 domain contributed in large part to this phenotype, but the V3V5 domain in this context did not. Interestingly, some of the V1V2 domains in subject 185F contained changes in the predicted glycosylation pattern relative to the 0-month Env, while others had sequence changes that would not alter the original glycosylation pattern (Fig. 7A: 5-PB1.1, 11-PL5.1, 23-PL5.1, and 28-PL5.1).

A novel finding is that Nab resistant Envs at 28-months utilized distinct Env sub-regions to block the same Nab pool. This provided a striking example of convergent, intra-patient evolution during early infection. One pathway was heavily dependent on the V5 domain, while the other exhibited V1V2 and gp41 co-dependence. The V5 domains of these two Envs contained the same predicted glycosylation shift (Fig. 7A: green and white spheres in 28-PL3.1 and 28-PL5.1) but differed in primary amino acid sequence (Fig. 4C). This raises the possibility that the V5-dependent Env contained mutations that directly confer epitope escape, while the V1V2-dependent Env retained the target but escaped through indirect mechanisms. In addition, both V1V2 and the regions flanking V5 are proximal to the CD4 binding site and could therefore alter its exposure, as has been proposed for changes in V2 and V5 in the context of a SHIV infection [30]. Thus, in examining a single subject in great detail, we have uncovered remarkable flexibility in the pathways of viral escape during early infection. These results further highlight how the plasticity of the Env hyper-variable domains coupled with complex conformational interactions could provide numerous options for escape.

### Different pathways were observed between subjects

**Figure 8:**
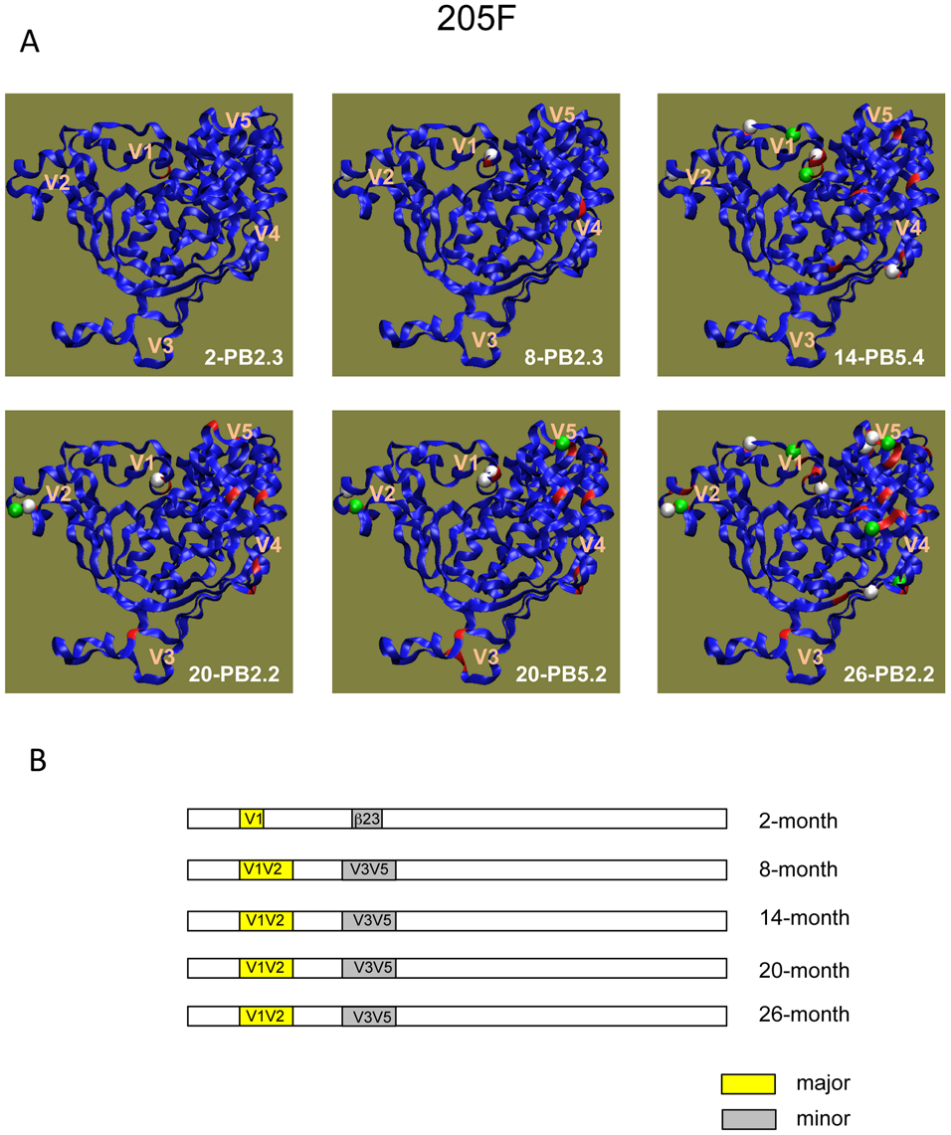
Nab escape in 205F is V1V2-dependent. (A) A 3-dimensional representation of the gp120 amino acid sequence for a different longitudinal Nab escape variant Env is shown in each panel. The time point and clone is indicated in each panel. The blue gp120 backbones were generated by homology modeling of the 0-month Env sequence onto the CD4-liganded HIV-1 YU-2 gp120 structure [54], with modeled V1V2 and V3 loops as described previously [25]. Red indicates amino acid changes relative to the 0-month Env sequence. Spheres indicate changes in a potential N-linked glycosylation sites (green = loss, white = gain). (B) Schematic representation of the Env domains contributing to Nab escape at each time point. Yellow indicates that the domain that had a major effect on Nab resistance; gray indicates the domain that made a minor contribution to Nab resistance. For more detail, see Fig. S5.

In contrast to subject 185F, Nab escape in 205F was driven predominantly by changes in the V1V2 domain (Fig. 8A and B). A preference for potential glycan shifts in V1V2 became evident from the spatial representation of gp120, where in the 14-month Env, four predicted glycosylation site changes are observed in V1 alone (Fig. 8A: green and white spheres in Env 14-PB5.4). The importance of V1V2 for escape was further illustrated by the demonstration that two predicted glycan sites, one in V1 and one in V2, influenced neutralization by two Mabs derived from this same subject. Thus, this study is the first to identify specific mutations that confer autologous Nab resistance at the single antibody level. This made possible several observations that were not apparent from polyclonal plasma. First, a single substitution can confer resistance against multiple antibody specificities within an individual. While we did not formally show that glycosylation at the substituted site was responsible for resistance against Mabs 6.4C and 13.6A, there is strong evidence from other studies to support that this is the case. Second, different pathways of escape also operate at the single antibody level. Resistance against 13.6A could be achieved either by addition of the predicted glycan site in V2 or by loss of the predicted glycan site in V1. Interestingly, only the modification of V2 was retained in subsequent escape variants, suggesting that it could have been more advantageous in terms of escape and or maintenance of replication fitness. Third, mutations that confer escape from multiple monoclonal antibody specificities do not necessarily confer escape from the entire polyclonal Nab milieu in plasma. The V2 modification in the 8-month Env conferred complete resistance against 6.4C and 13.6A at 10 µg/ml, but only partial escape from patient plasma (Fig. S5 and data not shown). This finding suggests that escape determinants mapped against plasma will only reflect the dominant Nab specificities that are present at relatively high concentration (able to inhibit virus infectivity at greater than a 1∶100 dilution in our assay), but other lower titer Nab specificities could also drive escape mutations. The relative contributions of different antibody specificities in plasma will undoubtedly vary among subjects, and potentially even within a subject over time, resulting in the need for customized escape pathways that are driven by each dominant Nab response. Thus, to derive a complete picture of autologous Nab and escape, it will be necessary to recover and characterize individual Mabs with different specificities, as was done here and recently by others to dissect the B cell response in subjects with neutralization breadth [9]. These studies demonstrate how the HIV-1 subtype C Env is uniquely equipped to respond to the current immune response of each individual host by adjusting its pathways of escape.

### Temporal properties of escape from autologous Mabs

The V2-based mutation that conferred resistance against 6.4C and 13.6A appeared during the first eight months of infection; however, 13.6A and 6.4C were recovered from memory B cells circulating 41 months later. As such, it was not possible to determine whether related B cells were circulating during early infection in 205F. The V1-based change that conferred resistance against 13.6A was present at 0-months (at which time the subject was seropositive), but only transiently. If this mutation occurred in response to immune pressure from 13.6A, then this antibody must have been present within ∼48 days from the calculated time of infection. Indeed we have observed very low level neutralizing activity (IC50 = ∼1∶40) in the 0-month plasma of 205F against 0-month Envs [15] and (data not shown), consistent with this concept. While others have observed that the very early antibody response (within the first ∼40 days after infection) lacks neutralizing activity and is directed predominantly against gp41 [40], our findings raise the possibility that viral neutralization and escape could occur earlier than previously thought. Individual Mabs, derived from B cells early in infection and tested against founder virus Envs, may be required to detect this initial Nab activity. It will therefore be important to determine whether Nab activity and viral escape is present in other subtype C infected subjects at very early time points, as well as whether early escape mutations are associated with decreased viral fitness.

### Spatial properties of escape pathways

**Figure 9:**
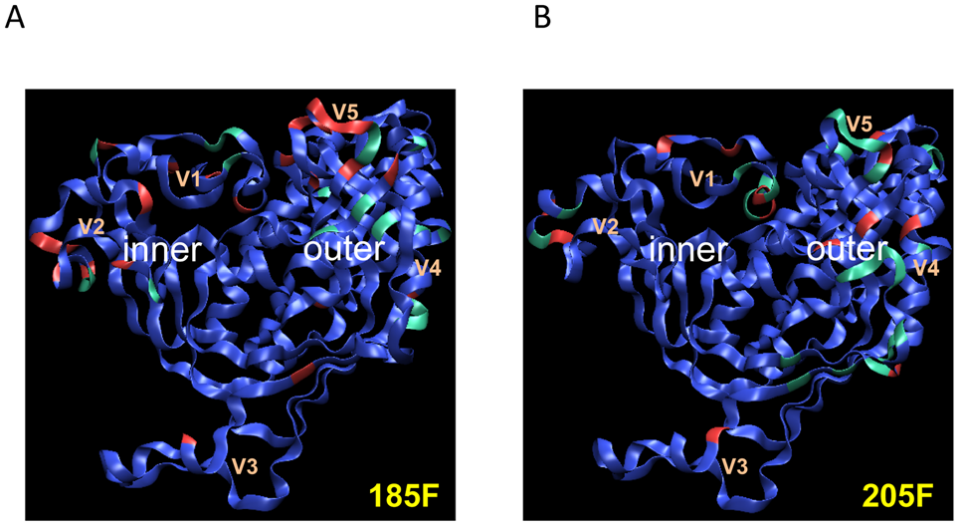
Sequence variation occurs in similar regions of Env in 185F and 205F. A 3-dimensional representation of the sequence variation in gp120 over time in 185F and 205F is shown in panels (A and B), respectively. The gp120 backbones were generated by homology modeling of the 0-month Env sequences of 185F and 205F onto the CD4-liganded HIV-1 YU-2 gp120 structure [54], with modeled V1V2 and V3 loops as described previously [25]. Blue to green to red indicates degree of sequence conservation (high to low) within the alignment.

Importantly in this study, not all sequence changes were linked directly with Nab escape. For example, one of the first sequence changes that was present in the 185F 5-month Env was a substitution in the first position of the α2 helix (E335A); however, this change did not alter Nab sensitivity to contemporaneous plasma when introduced into the 0-month Env. Reversal of this mutation (A335E) in the 5-month escape variant also did not increase its sensitivity to autologous Nab (Murphy et al., in preparation). The 205F Nab escape variants also exhibited variation in the α2 helix beginning at 14-months, but again this region did not appear to contribute independently to Nab escape. These findings support that α2 was not targeted directly by Nab in these instances, despite ongoing sequence evolution. The α2 helix has been linked to autologous and heterologous Nab sensitivity of subtype C Envs from Zambia, South Africa, and India by our group and others [23],[25],[41]. However, its exact role(s) in Nab sensitivity or escape remains undetermined [23],[25]. We have speculated that the α2 helix plays an ancillary role in Nab escape, and perhaps participates in maintenance of the tertiary structure of the gp120 outer domain or the quaternary structure of the trimer [25],[42]. The findings presented here support our earlier findings, but do not rule out the possibility that the α2 helix is targeted by Nab in some instances, or by low titer Nab specificities. Studies are ongoing in our laboratory to more precisely define the role of changes in the α2 helix in the context of autologous Nab. In addition to the α2 helix, sequence variation was observed in V1V2, V5, and other regions of the outer domain in both 185F and 205F Envs (Fig. 9A and B, respectively). However, in 185F, the V3V5 region had the strongest effect on Nab resistance, while in 205F V1V2 was the major determinant. A companion study of four subtype C infected seroconvertors and our own previous study of subtype C chronically infected subjects reported consistent findings, in that V1V2 was commonly involved in Nab escape, but to varying degrees [24] and (Moore et al., in press). Importantly, the combined biological results and spatial analysis of these two subjects demonstrate how the perpetual flexibility of the V1V2 and V5 domains provides a formidable defense against Nab. This could be due in part to their ability to simultaneously mask multiple epitopes through limited changes, but may also involve direct escape.

### Mechanistic properties of different escape pathways

Although these studies were conducted on a small number of subjects, it is still beneficial to work toward developing a mechanistic model that can explain the underlying complexity of escape pathways between and within subjects. The results presented here provide the basis for such an endeavor. First, the escape pathways observed here appear to define Nab resistance through a combination of direct and indirect mechanisms. Direct epitope changes may be sufficient in the setting of limited antibody specificities, such as during the early phase of infection, while indirect ‘masking’ or cooperative mechanisms may be required later when multiple antibody specificities are circulating. However, it will be important to expand and confirm these studies by characterizing Nab escape in additional subjects. The frequency, timing, and underlying basis for convergent escape pathways within a subject will require further investigation, as will the significance of regions of subtype C Env that appear to be under positive selection but do not contribute directly to escape from plasma Nab. Lastly, it will be important to determine if different escape pathways share any common conformational basis or point to specific regions of the Env that should be incorporated into or excluded from vaccine immunogens.

## Methods

### Ethics statement

Informed consent and human subjects protocols were approved by the Emory University Institutional Review Board, and the University of Zambia School of Medicine Research Ethics Committee. Written Informed consent was obtained from human subjects.

### Study subjects

The Zambia Emory HIV Research Project (ZEHRP) was established in Lusaka in 1994 to provide voluntary HIV-1 testing and counseling, long-term monitoring, and health care to cohabiting heterosexual couples. Details of the cohort have been described elsewhere [43]. Briefly, HIV-discordant couples enrolled in studies of transmission are monitored for seroconversion of the negative partner at three-month intervals, at which time the participants also receive preventative counseling and condoms. Banked plasma samples from seronegative partners are tested for p24 antigen by ELISA to identify individuals with acute infection [38]. The two subtype C infected seroconvertors studied here were participants in this cohort and were identified as p24 antigen positive and seropositive by rapid test and western blot, as described in [38],[44]. Plasma viral loads were determined using the Roche Amplicor HIV-1 assay. None of the subjects received antiretroviral therapy during the evaluation period.

### Amplification and cloning of HIV-1 *env* genes

Conditions for single genome PCR amplification of full-length gp160 (plus Rev, Vpu, and partial Nef coding sequences) from the genomic DNA of uncultured peripheral blood mononuclear cells and cDNA from plasma have been described previously [38],[44]. The viral *env* amplicons were directionally T/A cloned into the CMV-driven expression plasmid pcDNA3.1-V5HisTOPO-TA and screened for biological function as pseudoviruses following co-transfection with an Env-deficient subtype B proviral plasmid (SG3Δenv) into 293T cells [45]. Seventy-two hours later, supernatant was collected and used to infect JC53-BL13 (Tzm-bl) cells. At 48 hours post-infection, β-gal staining was performed and each well was scored positive or negative for blue foci.

### Sequence analysis

DNA sequencing of *env* genes was carried out by Lone Star Labs, Inc. (Houston, TX) utilizing the ABI Prism® Automated DNA sequencer 377XL and Big Dye™ Terminator Ready Reaction Cycle Sequencing Kit. Nucleotide sequences were edited and assembled using Sequencher v4.7, translated using Se-Al v2.0all, and nucleotide or amino acid alignments were created using Clustal W v1.83. Neighbor joining phylogenetic trees were generated by Clustal W v1.83 using gap-stripped nucleotide sequences of the complete *env* gene, and reliability of branching orders was assessed by bootstrap analysis using 1,000 replicates. Trees were visualized using NJ Plot. Aligned sequences were imported into the Highlighter tool to analyze viral diversity (http://www.hiv.lanl.gov/content/sequence/HIGHLIGHT/highlighter.html). Sequences have been deposited into Genbank under the accession numbers GQ485312-GQ485447.

### Neutralization assay

Patient plasma samples were evaluated for neutralizing antibody activity against virions pseudotyped with autologous patient-derived viral Envs using a single round reporter assay described previously [17],[45]. Briefly, JC53BL-13 (Tzm-bl) cells were plated and cultured overnight. Two thousand infectious units of each pseudovirus was combined with five-fold dilutions of heat-inactivated patient plasma and incubated for 1 hour at 37°C. Normal heat-inactivated human plasma was added as necessary to maintain a constant overall concentration. The virus-Ab mixture was then added to JC53BL-13 cells, and after two days, the cells were lysed, and the luciferase activity of each well was measured using a luminometer. Background luminescence was determined in uninfected wells and subtracted from all experimental wells. Percent infectivity was calculated by dividing the number of luciferase units at each plasma dilution by the value in the well containing no test plasma. The dilution of patient plasma that inhibited 50% of virus infectivity (IC50 titer) was determined using the Microsoft Excel 2004 for Mac Growth Function. Each experiment was performed independently at least twice with duplicate wells.

### Construction of chimeric Envs

Chimeric Envs were constructed using a domain exchange strategy that has been described previously [24],[25]. The primer sequences and their HXB2 locations are shown below. A PCR screen was performed to identify transformants in which the fragments ligated together in the correct orientation using forward primer EnvA and the reverse primer that was used to amplify the exchanged domain. Colonies that were positive by PCR screen were inoculated into LB-Ampicillin broth for overnight cultures, and the plasmid was prepared using the QIAprep Spin Miniprep Kit. Env chimeras were then screened for biological function as described above. For chimeras that produced functional Env pseudotypes, the plasmids were re-transfected into 293T cells on a larger scale to produce a working pseudotype virus stock. Transfection supernatants were collected at 72 hours post-transfection, clarified by low speed centrifugation, aliquoted into 0.5 ml or less portions, and stored at −80°C. The titer of each pseudovirus stock was determined by infecting JC53-BL13 cells with 5-fold serial dilutions of virus as described previously. All Env chimeras were confirmed by nucleotide sequencing of the entire *env* gene.

For subject 185F V1V5 domain amplification, forward primer 5′-catgtgtaaagttgaccccac-3′ (HXB2 nt 6577 to 6597) and reverse primer 5′-atctcctcctccaggtctgaa-3′ (HXB2 nt 7646 to 7626); V1V2 domain amplification, forward primer 5′-catgtgtaaagttgaccccac-3′ (HXB2 nt 6577 to 6597) and reverse primer 5′-tgttacggctgaggtattaca-3′ (HXB2 nt 6830 to 6810); V3V5 domain amplification, forward primer 5′-cccaacaataatacaaggaaa-3′ (HXB2 nt 7119 to 7139) and reverse primer 5′-atctcctcctccaggtctgaa-3′ (HXB2 nt 7646 to 7626); ecto domain amplification, forward primer 5′-agagcagtgggaataggagct-3′ (HXB2 nt 7755 to 7775) and reverse primer 5′-tatataccacagccatcttga-3′ (HXB2 nt 8270 to 8250); HR2 domain amplification, forward primer 5′-tgcaccactaatgtgccttgg-3′ (HXB2 nt 8034 to 8054) and reverse primer 5′-tatataccacagccatcttga-3′ (HXB2 nt 8270 to 8250); V5 domain amplification, forward primer 5′- taaatcaaatatcacaggact-3′ (HXB2 nt 7559 to 7579) and reverse primer 5′-atctcctcctccaggtctgaa-3′ (HXB2 nt 7646 to 7626).

For subject 205F, V1V5 domain amplification, forward primer 5′-gggatcaaagcctaaaaccat-3′ (HXB2 nt 6557 to 6579) and reverse primer 5′-cggtctgaatgtctctgt-3′ (HXB2 nt 7634 to 7617); V1V2 domain amplication, forward primer 5′-gggatcaaagcctaaaaccat-3′ (HXB2 nt 6557 to 6579) and reverse primer 5′-aataatgtataggaattggatc-3′ (HXB2 nt 6876 to 6855); V3V5 domain amplification, forward primer 5′-tgcacaagacccaacaataat-3′ (HXB2 nt 7110 to 7130) and reverse primer 5′-cggtctgaatgtctctgt-3′ (HXB2 nt 7634 to 7617).

The 0-month Env backbones (minus the target domains) with pcDNA3.1 vector sequences were PCR amplified using primers that anneal to conserved regions adjacent to the target domain primers. These primers amplify away from the target domains. A 5′ phosphate group (Phos) was added to these primer sets during synthesis to facilitate ligation to the target domain amplicons. The primer sequences and their HXB2 locations were as follows:

For subject 185F, V1V5 domain backbone, forward primer 5′-Phos-atgagggacaattggagaagtg-3′ (HXB2 nt 7647 to 7668) and reverse primer 5′-Phos-gctttaagctttgatcccataaac-3′ (HXB2 nt 6576 to 6553); V1V2 domain backbone, forward primer 5′-Phos-caagcctgtccaaaggtctct-3′ (HXB2 nt 6831 to 6851) and reverse primer 5′-Phos-gctttaagctttgatcccataaac-3′ (HXB2 nt 6576 to 6553); V3V5 domain backbone, forward primer 5′-Phos-atgagggacaattggagaagtg-3′ (HXB2 nt 7647 to 7668) and reverse primer 5′-Phos-ccttacacacacaatttctac-3′ (HXB2 nt 7118 to 7098); ectodomain backbone, forward primer 5′-Phos-aagatatttataatgatagta-3′ (HXB2 nt 8271 to 8291) and reverse primer 5′-Phos-tttttctctctccaccactctcc-3′ (HXB2 nt 7754 to 7732); V5 domain backbone, forward primer 5′-Phos-atgagggacaattggagaagtg-3′ (HXB2 nt 7647 to 7668) and reverse primer 5′-Phos-catgttatgtttcctgcaatg-3′ (HXB2 nt 4527 to 4507).

The PCR amplification conditions for the 0-month Env backbones were 1 cycle of 95°C for 3 min; 35 cycles of 95°C for 1 min, 50°C to 60°C for 30 s (the optimal annealing temperature was determined for each primer set), 72°C for 10 min; 1 cycle of 72°C for 15 min; and storage at 4°C. The amplification conditions for the target domains were the same, except the extension time at 72°C was reduced to 30 s. The 25 µl PCR mixtures contained 50 ng of each primer, 10 ng of the plasmid template, 2.5 mM MgCl_2_, 0.2 mM deoxynucleoside triphosphate, and 1× reaction buffer. *PfuUltra* II DNA polymerase (Stratagene) was used to generate the blunt-ended PCR amplicons, which were digested with DpnI to remove contaminating template DNA and gel purified from an agarose gel using the QIAquick Gel Extraction Kit (QIAGEN) prior to ligation. Each target domain DNA fragment was then ligated to the purified 0-month *env* backbone to produce a chimera using T4 DNA ligase (5 U/µl; Roche) at 4°C overnight. The ligation reaction mixture (usually one-third of the volume) was transformed into maximum-efficiency XL2-Blue Ultracompetent cells (1×10^9^ CFU/µg DNA; stratagene) so that the DNA volume did not exceed 5% of the cell volume. The entire transformation was plated onto LB-ampicillin agar plates, generally resulting in 10 to 50 colonies per ligation reaction.

### PCR-based site-directed mutagenesis

To investigate whether individual amino acid sequence differences contributed to the neutralization resistant phenotype, PCR-based site-directed mutagenesis was used to introduce an amino acid change as described previously [24]. Briefly, a set of primers was used that each had either the wildtype or mutated sequence. The primer sequences and their HXB2 locations are shown below, where the substituted nucleotides are underlined. All mutants were confirmed by sequencing the entire *env* gene.

The PCR amplification conditions were 1 cycle of 95°C for 1 min; 18 cycles of 95°C for 50 s, 60°C for 50 s, 68°C for 8 min; and 1 cycle of 68°C for 7 min. The 25-µl PCR mixtures contained 63 ng of each primer, 5 ng of the plasmid template, 0.2 mM deoxynucleoside triphosphate, and 1× reaction buffer. *PfuUltra* HF DNA polymerase (Stratagene) was used, amplicons were digested with DpnI to remove contaminating template DNA, and 2 µl was transformed into maximum-efficiency XL10-Gold ultracompetent cells (5×10^9^ CFU/µg DNA; Stratagene). Half of the transformation was plated onto LB-ampicillin agar plates, generally resulting in 10 to 50 colonies per reaction. All mutants were confirmed by sequencing the entire *env* gene.

185F E335A forward primer: 5′-gcatattgtaacattagtgcacagacatggaatgac-3′

185F E335A reverse primer: 5′-gtcattccatgtctgtgcactaatgttacaatatgc-3′ (HXB2 nt 7244 to 7209)

185F I459T forward primer: 5′-ctattgacacatgatgggacatatgcaaatagcaat-3′

185F I459T reverse primer: 5′-attgctatttgcatatgtcccatcatgtgtcaatag-3′ (HXB2 nt 7610 to 7575)

185F S463N forward primer: 5′-cacatgatgggatatatgcaaataacaataatacaacatta-3′

185F S463N reverse primer: 5′-taatgttgtattattgttatttgcatatatcccatcatgtg-3′ (HXB2 nt 7622 to 7582)

185F V518M forward primer: 5′-gggaataggagctatgttccttgggttcttggg-3′

185F V518M reverse primer: 5′-cccaagaacccaaggaacatagctcctattccc-3′ (HXB2 nt 7795 to 7763)

205F V1mut forward primer: 5′-gctgtagcaattatagcaattgtaatgatacc-3′

205F V1mut reverse primer: 5′-ggtatcattacaattgctataattgctacagc-3′ (HXB2 nt 6645 to 6614)

205F V2mut forward primer: 5′-gcctaatgatagtaactctagtgagtatatatta-3′

205F V2mut reverse primer: 5′-taatatatactcactagagttactatcattaggc-3′ (HXB2 nt 6812 to 6779)

### Generation of B cell hybridomas

Human B cell hybridomas were generated from viable frozen PBMC samples from subject 205F based on a protocol of EBV immortalization of peripheral blood B cells as described previously [46],[47],[48]. This protocol includes immortalization of pre-selected (CD22+, IgM−, IgD−, IgA−) memory B cell populations that are cultured in medium supplemented with immunostimulatory CpG sequences and irradiated allogeneic PBMC, as described in [49],[50],[51]. EBV-transformed B cell cultures were screened for Mabs that neutralized the 205F 0-month Env and that bound envelope glycoproteins by ELISA [52],[53]. The method used to screen for neutralizing Mabs is a modification of the Tzm-bl (JC53-BL13) luciferase reporter cell assay originally developed by Wei et al. [15],[17],[24],[45]. EBV-transformed B cell hybridomas with neutralizing activity were cloned in the presence of CpG and irradiated PBMC. Culture supernatant was collected from the two hybridomas, 6.4C and 13.6A, and used in the neutralization studies to map targets and escape.

## Supporting Information

Figure S1 Phylogenetic tree of longitudinal 185F and 205F Envs. A. A neighbor-joining tree was generated using Clustal W v.1.83 for OSX using the entire *env* nucleotide sequence of 103 185F Envs from 11 time points and 8 Envs from the donor partner 185M at 0-months. A consensus C sequence was used to root the tree. An asterisk indicates a bootstrap greater than 80. Closed symbols indicate Envs from plasma; open symbols indicate Envs from uncultured PBMC DNA. Colors from warm to cool indicate longitudinal time points; squares indicate Envs from the donor partner. A red arrow indicates the two 0-month Envs (PL3.1 and PB3.1) that were used as a background for chimeras and mutants; black arrows indicate Nab resistant Envs used for domain exchange into the chimera. Shaded gray boxes indicate 3 distinct phylogenetic lineages that included 28-month escape variants. B. A neighbor-joining tree was generated using Clustal W v.1.83 for OSX using the entire *env* nucleotide sequence of 34 205F Envs from 7 time points and 5 Envs from the donor partner 205M at 0-months. A consensus C sequence was used to root the tree. An asterisk indicates a bootstrap greater than 80. Closed symbols indicate Envs from plasma; open symbols indicate Envs from uncultured PBMC DNA. Colors from warm to cool indicate longitudinal time points; squares indicates Envs from the donor partner. A red arrow indicates the 0-month Env (PL6.3) that was used as a background to construct chimeras; black arrows indicate Nab resistant Envs used for domain exchange into the chimera. Shaded gray boxes indicate 2 different Nab resistant Envs from the 20-month time point.
(0.79 MB PDF)

Figure S2 Amino acid sequence alignment for 205F Envs. Three 0-month Nab sensitive Envs and six Nab resistant Envs from subsequent time points were selected for study from 32 Envs. Env clones are indicated by the time point (in months), source (PB = PBMC DNA or PL = plasma), and clone number. Sequences are shown in reference to the 0-month EnvPB1.1, with amino acid differences indicated by the letter, and deleted residues indicated by a dot. Domains that were transferred into the 0-month Env to create chimeras are as follows: V1V5 (blue, gray and green; HXB2 nt 6557 to 7634), V1V2 (blue; HXB2 nt 6557 to 6876), V3V5 (green; HXB2 nt 7110 to 7634). Major Env domains are indicated above the region, and the α2 helix is underlined. Two potential N-linked glycan addition sites of interest in V1 and V2 (NXS or NXT where X is any residue but proline) are highlighted yellow.
(0.37 MB PDF)

Figure S3 Amino acid sequence alignment for 185F Envs. Two 0-month Nab sensitive Envs and ten Nab resistant Envs from subsequent time points were selected for study from 58 Envs. Env clones are indicated by time point (in months), the source (PB = PBMC DNA or PL = plasma), and clone number. Sequences are shown in reference to the 0-month Env PL3.1, with amino acid differences indicated by the letter, and deleted residues indicated by a dot. Domains that were transferred into the 0-month Env to create chimeras are as follows: V1V5 (blue, gray, and green; HXB2 nt 6577 to 7646), V1V2 (blue; HXB2 nt 6577 to 6810), V3V5 (green; HXB2 nt 7119 to 7646), gp41 ectodomain (yellow; HXB2 nt 7755 to 8270).
(0.50 MB PDF)

Figure S4 The major determinants of Nab resistance in 185F Envs change over time. Neutralization of 185F parental and chimeric Env pseudoviruses was evaluated using longitudinal plasma samples that are indicated in each panel. Each plasma sample was contemporaneous with the Nab resistant Env, and neutralization sensitivity was evaluated in JC53-BL cells using luciferase as a quantitative measure. Percent virus infectivity is plotted against the reciprocal of the log10 reciprocal plasma dilution. Error bars represent the standard deviation of at least two independent experiments using duplicate wells. All chimeras were created in the 0-month EnvPL3.1 background (red lines). In the legend, the parental Env clones are indicated, followed by each chimera that contains the indicated region from the Nab resistant Env in the 0-month Env. ‘ecto’ stands for the gp41 ectodomain.
(0.22 MB PDF)

Figure S5 The major determinants of Nab resistance in 205F Envs are in V1V2. Neutralization of 205F parental and chimeric Env pseudoviruses was evaluated using longitudinal plasma samples that are indicated in each panel. Each plasma sample was contemporaneous with the Nab resistant Env, and neutralization sensitivity was evaluated in JC53-BL cells using luciferase as a quantitative measure. Percent virus infectivity is plotted against the reciprocal of the log10 reciprocal plasma dilution. Error bars represent the standard deviation of at least two independent experiments using duplicate wells. All chimeras were created in the 0-month EnvPL6.3 background (red lines). In the legend, the parental Env clones are indicated, followed by each chimera that contains the indicated region from the Nab resistant Env in the 0-month Env.
(0.14 MB PDF)

Figure S6 Highlighter plot showing nucleotide mismatches in the gp120 V1V4 region for 205F Envs. Single genome amplified, uncloned V1V4 sequences derived from the 1-Mar-03 sample (n = 21) or the 27-Mar-03 sample (n = 31) from 205F and V1V4 sequences from single genome amplified cloned Envs from the 27-Mar-03 sample (n = 5) were subjected to Highlighter analysis (www.hiv.lanl.gov). Ticks represent mismatched bases compared to the master sequence listed at the top. The three 0-month Envs that were analyzed for neutralization by 6.4C and 13.6A in Fig. 11 are boxed in red. PB = uncultured PBMC DNA; PL = plasma. The G>A mutation (diamonds) located near the beginning of the sequence represents the loss of a potential N-gly site in V1 that tracks with resistance against 13.6A.
(0.11 MB PDF)
