# Supplementary figures and images for: Escape from Autologous Neutralizing Antibodies in Acute/Early Subtype C HIV-1 Infection Requires Multiple Pathways
Source: PLoS Pathog. 2009 Sep 18;5(9):e1000594. doi: 10.1371/journal.ppat.1000594 (PMC2741593; doi:10.1371/journal.ppat.1000594)

A. 185F

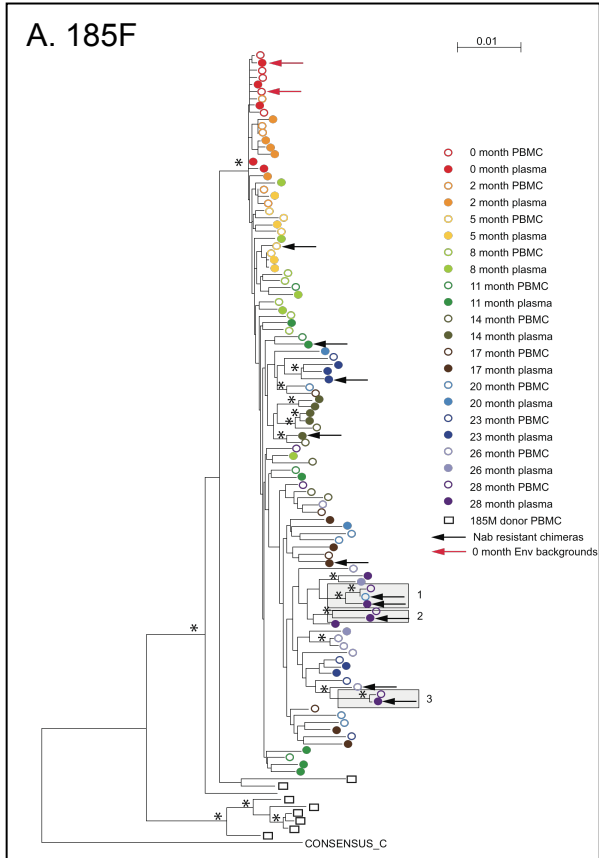

B. 205F

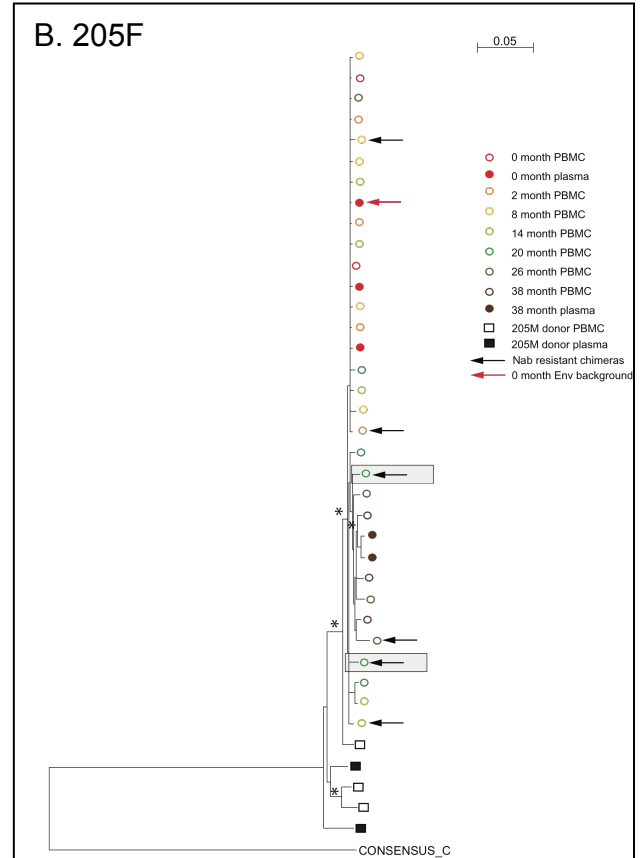

Supplement: Figure S1 — Phylogenetic tree of longitudinal 185F and 205F Envs. A. A neighbor-joining tree was generated using Clustal W v.1.83 for OSX using the entire env nucleotide sequence of 103 185F Envs from 11 time points and 8 Envs from the donor partner 185M at 0-months. A consensus C sequence was used to root the tree. An asterisk indicates a bootstrap greater than 80. Closed symbols indicate Envs from plasma; open symbols indicate Envs from uncultured PBMC DNA. Colors from warm to cool indicate longitudinal time points; squares indicate Envs from the donor partner. A red arrow indicates the two 0-month Envs (PL3.1 and PB3.1) that were used as a background for chimeras and mutants; black arrows indicate Nab resistant Envs used for domain exchange into the chimera. Shaded gray boxes indicate 3 distinct phylogenetic lineages that included 28-month escape variants. B. A neighbor-joining tree was generated using Clustal W v.1.83 for OSX using the entire env nucleotide sequence of 34 205F Envs from 7 time points and 5 Envs from the donor partner 205M at 0-months. A consensus C sequence was used to root the tree. An asterisk indicates a bootstrap greater than 80. Closed symbols indicate Envs from plasma; open symbols indicate Envs from uncultured PBMC DNA. Colors from warm to cool indicate longitudinal time points; squares indicates Envs from the donor partner. A red arrow indicates the 0-month Env (PL6.3) that was used as a background to construct chimeras; black arrows indicate Nab resistant Envs used for domain exchange into the chimera. Shaded gray boxes indicate 2 different Nab resistant Envs from the 20-month time point. (0.79 MB PDF) [file ppat.1000594.s001.pdf]

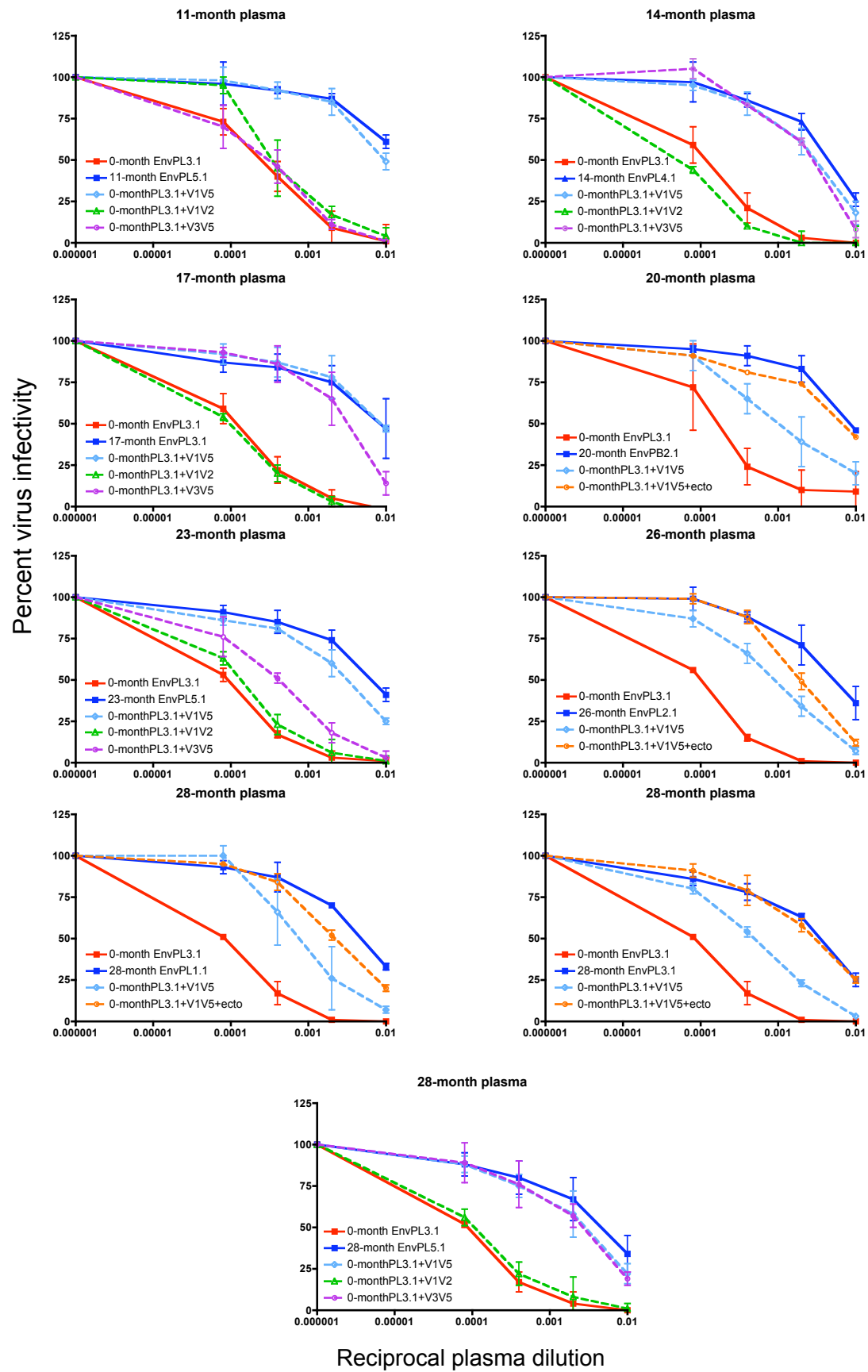

Supplement: Figure S4 — The major determinants of Nab resistance in 185F Envs change over time. Neutralization of 185F parental and chimeric Env pseudoviruses was evaluated using longitudinal plasma samples that are indicated in each panel. Each plasma sample was contemporaneous with the Nab resistant Env, and neutralization sensitivity was evaluated in JC53-BL cells using luciferase as a quantitative measure. Percent virus infectivity is plotted against the reciprocal of the log10 reciprocal plasma dilution. Error bars represent the standard deviation of at least two independent experiments using duplicate wells. All chimeras were created in the 0-month EnvPL3.1 background (red lines). In the legend, the parental Env clones are indicated, followed by each chimera that contains the indicated region from the Nab resistant Env in the 0-month Env. ‘ecto’ stands for the gp41 ectodomain. (0.22 MB PDF) [file ppat.1000594.s004.pdf]

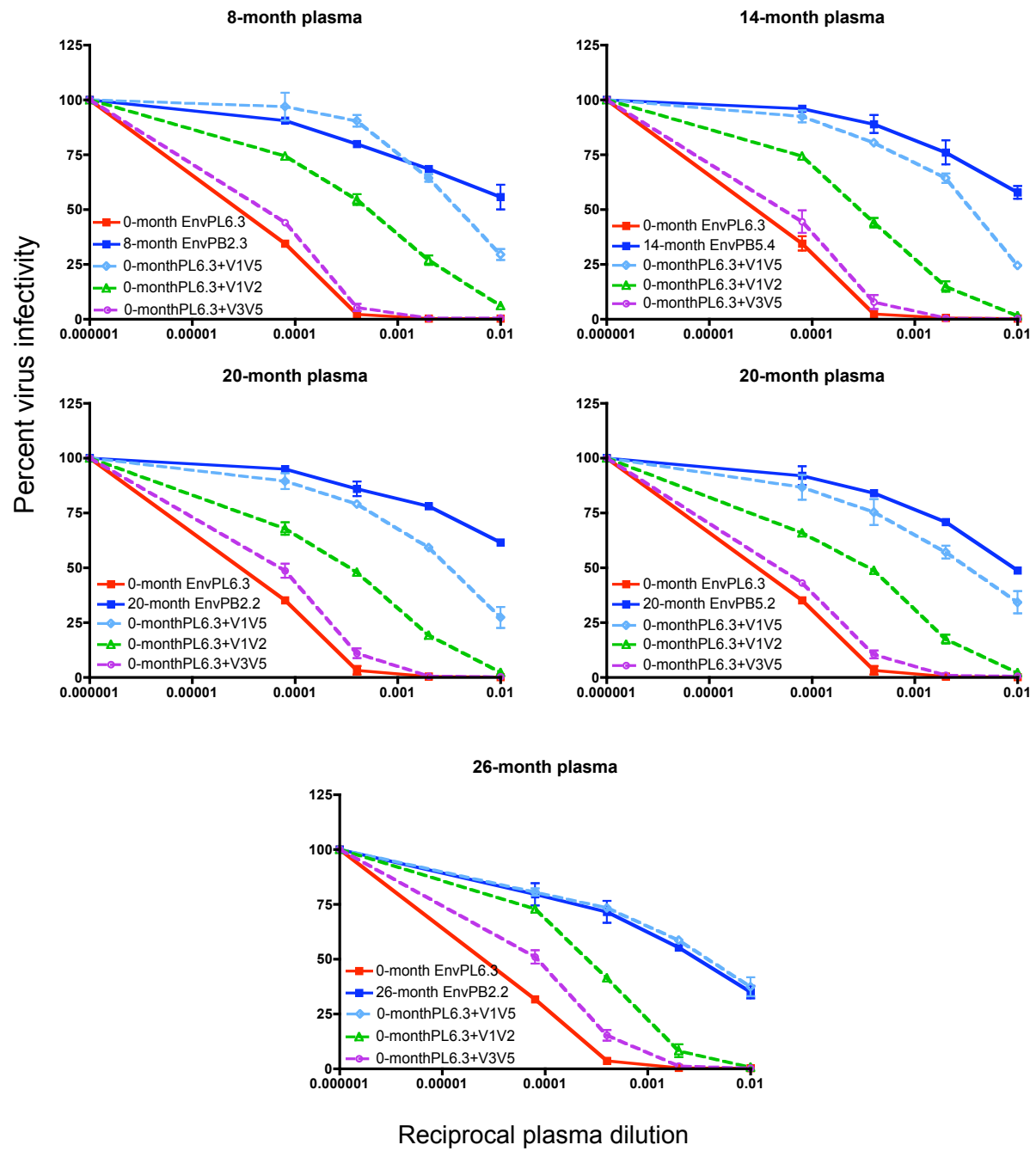

Supplement: Figure S5 — The major determinants of Nab resistance in 205F Envs are in V1V2. Neutralization of 205F parental and chimeric Env pseudoviruses was evaluated using longitudinal plasma samples that are indicated in each panel. Each plasma sample was contemporaneous with the Nab resistant Env, and neutralization sensitivity was evaluated in JC53-BL cells using luciferase as a quantitative measure. Percent virus infectivity is plotted against the reciprocal of the log10 reciprocal plasma dilution. Error bars represent the standard deviation of at least two independent experiments using duplicate wells. All chimeras were created in the 0-month EnvPL6.3 background (red lines). In the legend, the parental Env clones are indicated, followed by each chimera that contains the indicated region from the Nab resistant Env in the 0-month Env. (0.14 MB PDF) [file ppat.1000594.s005.pdf]

205F

### Sequences compared to master

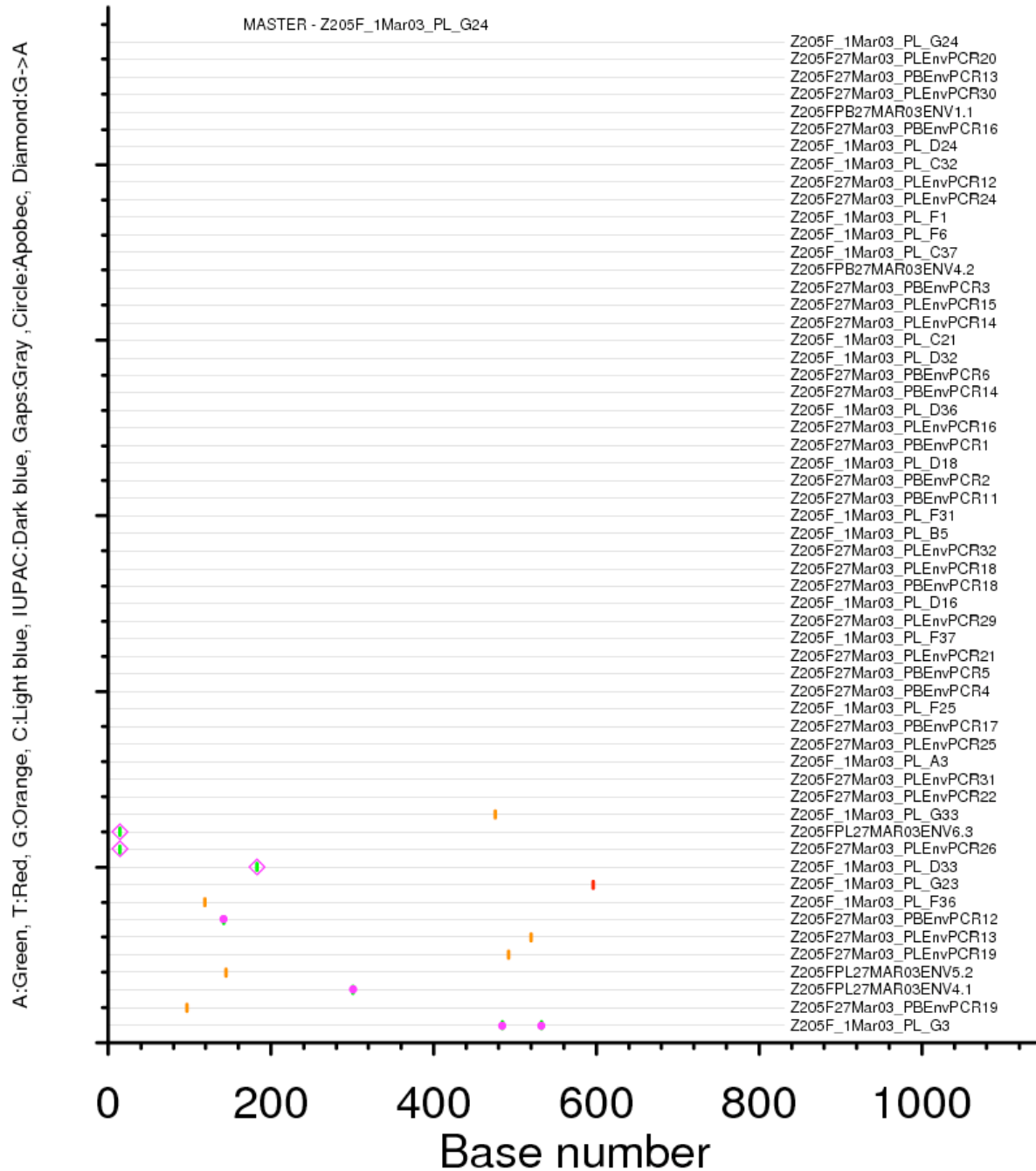

Supplement: Figure S6 — Highlighter plot showing nucleotide mismatches in the gp120 V1V4 region for 205F Envs. Single genome amplified, uncloned V1V4 sequences derived from the 1-Mar-03 sample (n = 21) or the 27-Mar-03 sample (n = 31) from 205F and V1V4 sequences from single genome amplified cloned Envs from the 27-Mar-03 sample (n = 5) were subjected to Highlighter analysis (www.hiv.lanl.gov). Ticks represent mismatched bases compared to the master sequence listed at the top. The three 0-month Envs that were analyzed for neutralization by 6.4C and 13.6A in Fig. 11 are boxed in red. PB = uncultured PBMC DNA; PL = plasma. The G>A mutation (diamonds) located near the beginning of the sequence represents the loss of a potential N-gly site in V1 that tracks with resistance against 13.6A. (0.11 MB PDF) [file ppat.1000594.s006.pdf]
