# Supplementary material for: Escape from Autologous Neutralizing Antibodies in Acute/Early Subtype C HIV-1 Infection Requires Multiple Pathways
Source: PLoS Pathog. 2009 Sep 18;5(9):e1000594. doi: 10.1371/journal.ppat.1000594 (PMC2741593; doi:10.1371/journal.ppat.1000594)
Supplement: Figure S2 — Amino acid sequence alignment for 205F Envs. Three 0-month Nab sensitive Envs and six Nab resistant Envs from subsequent time points were selected for study from 32 Envs. Env clones are indicated by the time point (in months), source (PB = PBMC DNA or PL = plasma), and clone number. Sequences are shown in reference to the 0-month EnvPB1.1, with amino acid differences indicated by the letter, and deleted residues indicated by a dot. Domains that were transferred into the 0-month Env to create chimeras are as follows: V1V5 (blue, gray and green; HXB2 nt 6557 to 7634), V1V2 (blue; HXB2 nt 6557 to 6876), V3V5 (green; HXB2 nt 7110 to 7634). Major Env domains are indicated above the region, and the α2 helix is underlined. Two potential N-linked glycan addition sites of interest in V1 and V2 (NXS or NXT where X is any residue but proline) are highlighted yellow. (0.37 MB PDF) [file ppat.1000594.s002.pdf]

205F

```
 0-PB1.1 MRVMGMSRNY QQWWIWGILG FWMLMICSVM GNLWVTVYYG VPVWREAKTT LFCASDAKAY EREVHNVWAT HACVPTDPNP QEMFLKNVTE NFNMWKNDMV  100
 0-PL4.1 ---------- ---------- ---------- ---------- ---------- ---------- ---------- ---------- ---------- ----------
 0-PL6.3 ---------- ---------- ---------- ---------- ---------- ---------- ---------- ---------- ---------- ----------
 2-PB2.3 ---------- ---------- ---------- ---------- ---------- ---------- ---------- ---------- ---------- ----------
 8-PB2.3 ---------- ---------- ---------- ---------- --------P- ---------- ---------- ---------- ---------- ----------
14-PB5.4 ---------- -------T-- ---------- ---------- ---------- ---------- ---------- ---------- ---------- ----------
20-PB2.2 ---------- -------V-- --------GV ---------- ---------- ---------- ---------- ---------- -----E---- D---------
20-PB5.2 ---------- ---------- --------G- ---------- ---------- ---------- ---------- ---------- -----E---- ----------
26-PB2.2 ---------- ---------- --------G- ---------- ---------- ---------- ---------- ---------- ---------- D---------

                                                           V1                                                  V2
 0-PB1.1 DQMHEDIISL WDQSLKPCVK LTPLCVTLSC SNYSNCNDTY S......... .TANCTSGGE IKNCSFNATT EIRDKNRKEY ALFYRPDIVP LKPNDSNSRE  200
 0-PL4.1 --------N- ---------- ---------- ---------- -......... .--------- ---------- ---------- ---------- ----------
 0-PL6.3 ---------- ---------- ---------- ---N------ -......... .--------- ---------- ---------- ---------- ----------
 2-PB2.3 ---------- ---------- ---------- ---------P -......... .--------- ---------- ---------- ---------- ----------
 8-PB2.3 ---------- ---------- ---------- ---------- -N.....DTN SI-------- ---------- ---------- ---------- --------S-
14-PB5.4 ---------- ---------- --------N- -D------NS T......... ..D------- ---------- ---------- ---------- --------S-
20-PB2.2 ---------- ---------- ---------- ---------D -N...YNKTY NK-------- ---------- ---------- ---------- -----N--S-
20-PB5.2 ---------- ---------- ---------- ---------- -NGTYSNDTN S-V----R-- ---------- ---------- ---------- ----EG--S-
26-PB2.2 ---------- ---------- --------N- -D---G-G-N N......... .---Y----- ---------- ---------- ---------- -----NK-S-

 0-PB1.1 YILINCNTST IAQACPKVSF DPIPIHYCAP AGYAILKCND NKTFNGTGPC HNVSTVQCTH GIKPVISTQL LLNGSLAEED IIIRSENLAN NVKTIIVHLN  300
 0-PL4.1 ---------- ---------- ---------- ---------N ---------- ---------- ---------- ---------- ---------- ----------
 0-PL6.3 ---------- ---------- ---------- ---------- ---------- ---------- ---------- ---------- ---------- ----------
 2-PB2.3 ---------- ---------- ---------- ---------- ---------- ---------- ---------- ---------- ---------- ----------
 8-PB2.3 ---------- ---------- ---------- ---------- ---------- ---------- ---------- ---------- ---------- ----------
14-PB5.4 ---------- ---------- ---------- ---------- ---------- ---------- ---------- ---------- ---------- ----------
20-PB2.2 ---------- ---------- ---------- ---------- ---------- ---------- ---------- ---------- ---------- ----------
20-PB5.2 ---------- ---------- ---------- ---------- ---------- R--------- ---------- ---------- ---------- ----------
26-PB2.2 -L-------- ---------- ---------- ---------- ---------- R--------- ---------- ---------- --------T- ----------

                        V3                                                α2
 0-PB1.1 ESVEINCTRP NNNTSRGIRI GPGQTFFATG RIIGNIRQAY CSINKSKWND TLQKIKGKLQ EHFPNKTIHF EPHSGGDLEI TTHSFNCRGE FFYCNTSELF  400
 0-PL4.1 ---------- ---------- ---------- ---------- ---------- ---------- ---------- ---------- ---------- ----------
 0-PL6.3 ---------- ---------- ---------- ---------- ---------- ---------- ---------- ---------- ---------- ----------
 2-PB2.3 ---------- ---------- ---------- ---------- ---------- ---------- ---------- ---------- ---------- ----------
 8-PB2.3 ---------- ---------- ---------- ---------- ---------- ---------- ---------- ---------- ---------- ----------
14-PB5.4 ---------- ---------- ---------- ---------- ----R----- -----R---- ---------- K--------- ---------- ----------
20-PB2.2 ---------- ---------- ---------- ----D----- ---------- -----E---- --------Q- ---------- ---------- ----------
20-PB5.2 ---------- ------SV-- ---------- ----D----- ----R----- -----E---- --------D- ---------- ---------- ----------
26-PB2.2 ---------- ---------- ---------- ----D----H -N-T------ ---R------ --------Q- K--------- ---------- ----------

              V4                                                             V5
 0-PB1.1 NISRLNSTSS IITLPCRIKQ FINMWQKVGR AMYAPPIEGK ITCNSSITGL LLTRDGGNNT NNTETFRPGG GDMRDNWRSE LYKYKVVEIK PLGIAPTGSK  500
 0-PL4.1 ---------- ---------- ---------- ---------- ---------- ---------- ---------- ---------- ---------- ----------
 0-PL6.3 ---------- ---------- ---------- ---------- ---------- --A------- ---------- ---------- ---------- ----------
 2-PB2.3 ---------- ---------- ---------- ---------- ---------- ---------- ---------- ---------- ---------- ----------
 8-PB2.3 ---G------ ---------- ---------- ---------- ---------- ---------- ---------- ---------- ---------- ----------
14-PB5.4 ---------- N---T----- ---------- ---------- ---------- ---------- -D-------- ---------- ---------- ----------
20-PB2.2 ------K--P ---------- ---------- ---------- ---------- --------T- ---------- ---------- ---------- ----------
20-PB5.2 ---------P ---------- ---------- ---------- ---------- ---------- ST-------- ---------- ---------- ----------
26-PB2.2 ..T------P ---------- ---------- ---------- ---------- ---------S S.N------- ---------- ---------- ----------

 0-PB1.1 RAVVEREKRA VGLGAVFLGF LGAAGSTMGA ASITLTVQAR QVLAGIVQQQ SNLLRAIEAQ QHMLQLTVWG IKQLQARVLA IERYLKDQQI LGIWGCSGKL  600
 0-PL4.1 ---------- ---------- ---------- ---------- ---------- ---------- ---------- ---------- ---------- ----------
 0-PL6.3 ---------- ---------- ---------- ---------- ---------- ---------- ---------- ---------- ---------- ----------
 2-PB2.3 ---------- ---------- ---------- ---------- ---------- ---------- ---------- ---------- ---------- ----------
 8-PB2.3 ---------- ---------- ---------- ---------- ---------- ---------- ---------- ---------- ---------- ----------
14-PB5.4 ---------- ---------- ---------- ---------- ---------- ---------- ---------- ---------- ---------- ----------
20-PB2.2 ----K----- ---------- ---------- ---------- ---------- ---------- ---------- ---------- ---------- ----------
20-PB5.2 ----Q----- ---------- ---------- ---------- ---------- ---------- ---------- ---------- ---------- ----------
26-PB2.2 ----K----- ---------- ---------- ---------- ---------- -S-------- ---------- ---------- ---------- ----------

 0-PB1.1 ICTTAVPWNA SWSNKSQEDI WNNTTWRQWD REISNYTSII YNLLEESQNQ QERNEKDLLA LDSWKNLWNW FDISRWLWYI KIFIIIVGGL IGLRIIFAVL  700
 0-PL4.1 ---------- ---------- ---------- ---------- ---------- ---------- ---------- ---------- ----M----- ----------
 0-PL6.3 ---------- ---------- ---------- ---------- ---------- ---------- ---------- ---------- ----M----- ----------
 2-PB2.3 ---------- ---------- ---------- ---------- ---------- ---------- ---------- ---------- ----M----- ----------
 8-PB2.3 ---------- ---------- ---M------ ---------- ---------- ---------- ---------- ---------- ----M----- ----------
14-PB5.4 ---------- ---------- ---M--M--- ---------- ---------- ---------- ---------- ---------- ----M----- ----------
20-PB2.2 ---------- ---------- ---M--M--- ---------- ---------- ---------- ---------- ---------- ----M----- ----------
20-PB5.2 ---------- ---------- ---M--M--- ---------- ---------- ---------- ---------- ---------- ----M----- ----------
26-PB2.2 ---------- -------D-- ---M--M--- ---------- ---------- ---------- ---------- -N-------- ----M----- ----------

 0-PB1.1 SIVNRVRQGY SPLSFQTLTP NPRELDRLGR VEEEGGEQDR DKSIRLVNGF LALFWDDLRS LCLFSYHHLR DFILIAARVV ELLGRRGWEA LKYLGSLVQY  800
 0-PL4.1 ---------- ---------- ---------- I--------- ---------- ---------- ---------- ---------- ---------- ----------
 0-PL6.3 ---------- ---------- ---------- I--------- ---------- ---------- ---------- ---------- ---------- ----------
 2-PB2.3 ---------- ---------- ---------K I--------- ---------- ---------- ---------- ---------- ---------- ----------
 8-PB2.3 ---------- ---------- ---------- I--------- ---------- ---------- ---------- --V------- ---------- ----------
14-PB5.4 ---------- ---------- ---------K I--------- ---------- ---------- ---------- ---------- ---------- ----------
20-PB2.2 ---------- ---------- ---------- I--------- ---------- ---------- ---------- ---------- ---------- ----------
20-PB5.2 ---------- ---------- ---------- I--------- ---------- ---------- ---------- ---------- ---------- ----------
26-PB2.2 ---------- ---------- ---------- I--------- ---------- ---------- ---------- ---------- ---------- ----------

 0-PB1.1 WGLELKKSAI SLFDTIAIAV AEGTDRIIEL IQRFGRGILN IPTRIRQGLE AALQ 854
 0-PL4.1 ---------- ---------- ---------- ---------- ---------- ----
 0-PL6.3 ---------- ---------- ---------- ---------- ---------- ----
 2-PB2.3 ---------- ---------- --------D- ---------- ---------- ----
 8-PB2.3 ---------- ---------- ---------- ---------- ---------- ----
14-PB5.4 ---------- --L------- ---------- ---------- ---------- ----
20-PB2.2 ---------- --L------- ---------- ---------- ---------- ----
20-PB5.2 ---------- --L------- ---------- ---------- -L-------- ----
26-PB2.2 ---------- --L------- ---------- ---------- ---------- ----
```
