# Supplementary material for: Escape from Autologous Neutralizing Antibodies in Acute/Early Subtype C HIV-1 Infection Requires Multiple Pathways
Source: PLoS Pathog. 2009 Sep 18;5(9):e1000594. doi: 10.1371/journal.ppat.1000594 (PMC2741593; doi:10.1371/journal.ppat.1000594)
Supplement: Figure S3 — Amino acid sequence alignment for 185F Envs. Two 0-month Nab sensitive Envs and ten Nab resistant Envs from subsequent time points were selected for study from 58 Envs. Env clones are indicated by time point (in months), the source (PB = PBMC DNA or PL = plasma), and clone number. Sequences are shown in reference to the 0-month Env PL3.1, with amino acid differences indicated by the letter, and deleted residues indicated by a dot. Domains that were transferred into the 0-month Env to create chimeras are as follows: V1V5 (blue, gray, and green; HXB2 nt 6577 to 7646), V1V2 (blue; HXB2 nt 6577 to 6810), V3V5 (green; HXB2 nt 7119 to 7646), gp41 ectodomain (yellow; HXB2 nt 7755 to 8270). (0.50 MB PDF) [file ppat.1000594.s003.pdf]

185F

```
 0-PL3.1 MRVMGTSRNC QLWWIWGILG FWMLMICNAE EKSWVTVYYG VPVWKEAKAP LFCASDAKAY EREVHNVWAT HACVPTDPNP QEIILKNVTE NFNMWKNDMV  100
 0-PB3.1 ---------- ---------- ---------- ---------- ---------- ---------- ---------- ---------- ---------- ----------
 5-PB1.1 ----EI---- ---------- -------D-- ---------- ---------- ---------- ---------- ---------- ---------- ----------
11-PL5.1 ----E-L--- ---------- ---------- --L------- ---------- ---------- ---------- ---------- -----E---- ----------
14-PL4.1 -----I---- -P-------- ---------- ---------- ---------- ---------- ---------- ---------- ---------- ----------
17-PL3.1 ------L-S- -P-------- ---------- ---------- ---------- ---------- ---------- ---------- --M------- ---------A
20-PB2.1 -----I---- -P-------- ---------- --L------- ---------- ---------- ---------- ---------- --MT------ ----------
23-PL5.1 -----I---- -P-------- -------K-- --L------- ---------- ---------- ---------- ---------- ---------- ----------
26-PL2.1 ----E----- -P-------- ---------- -ET------- ---------- ---------- ---------- ---------- --M------- ----------
28-PL1.1 -----I---- -P-------- ---------- --L------- ---------- ---------- ---------- ---------- --MT------ ----------
28-PL3.1 ----E----- -P-------- ---------- --L------- ---------- ---------- ---------- ---------- --MV------ ---------A
28-PL5.1 ----E----- -P-------- ---------- --L------- ---------- ---------- ---------- ---------- --MS------ ----------

                                                V1                                             V2
 0-PL3.1 DQMNEDIISL WDQSLKPCVK LTPLCVTLNC SDYNSTANRT ESTNNTSADI KQEIKNCSFY TTTEIKDKKK AEYALFYKLD IVSLYGNESS NKYRLINCNT  200
 0-PB3.1 ---------- ---------- ---------- ---------- ---------- ---------- ---------- ---------- ---------- ----------
 5-PB1.1 ---------- ---------- ---------- ---------- ---------- ---------- ---------- ---------- ---------- -Q--------
11-PL5.1 ---------- ---------- ---------- ----N---C- ---------- ---------- ---------- ---------- ----N--G-- KQ--------
14-PL4.1 ---------- ---------- ---------- --------S- ---------- ---------- ---------- ---------- ----N-S--- KQ--------
17-PL3.1 ---------- ---------- ---------- -N------H- ---------L ---------- -----Q---- ---------- ----D--N-- -T--------
20-PB2.1 ---------- ---------- ---------- --------S- ----.----V -----K---N I----Q---- T-H------- ----D--GN- TQ--------
23-PL5.1 ---------- ---------- ---------- -N--I---H- ---------L ---------- ---------- ---------- ----P--K-. .Q--------
26-PL2.1 ---------- ---------- ---------- -NF-----H- ---------L -----R---N -----Q---- T--------- ----D---N- TQ--------
28-PL1.1 ---------- ---------- ---------- --------S- ----.----V -----K---N I----Q---- T-H------- ----D--GN- TQ--------
28-PL3.1 ---------- ---------- ---------- -NF-----H- ---------- ---------- -----Q---- ----F----- ----P--K.. SQ--------
28-PL5.1 ---------- ---------- ---------- -NF-----H- ---------S -----R---N -----Q---- T-H------- ----D---N- TQ--------

 0-PL3.1 SAVTQACPKV SFDPIPIHYC APAGYAILKC NNNTFNGTGP CNNVSTVQCT HGITPVVSTQ LLLNGSLAEK EIIIRSENLT DSVKTIIVQF NKSVEIVCVR  300
 0-PB3.1 ---------- ---------- ---------- ---------- ---------- ---K------ ---------- ---------- ---------- ----------
 5-PB1.1 ---------- ---------- ---------- ---------- ---------- ---K------ ---------- ---------- ---------- ----------
11-PL5.1 ---------- ---------- ---------- ---------- ---------- ---K------ ---------- ---------- ---------- ----------
14-PL4.1 ---------- ---------- ---------- ---------- ---------- ---K------ ---------- ---------- -----M---- ----------
17-PL3.1 ---------- ---------- ---------- ---------- ---------- ---K------ ---------- ---------- -N-------- --P-------
20-PB2.1 ---------- ---------- ---------- ---------- ---------- ---K------ ---------E ---------- -N-------- --P-------
23-PL5.1 ---------- ---------- ---------- ---------- --S------- ---K------ ---------- ---------- N--------- ----------
26-PL2.1 ---------- ---------- ---------- ---------- ---------- ---K------ ---------- ---------- -N-------- --P-------
28-PL1.1 ---------- ---------- ---------- ---------- ---------- ---K------ ---------E ---------- -N-------- --P-------
28-PL3.1 ---------- ---------- ---------- ---------- ---------- ---K------ ---------E ---------- -N-------- -EP-------
28-PL5.1 ---------- ---------- ---------- ---------- --D------- ---K------ ---------- ---------- ---------- -E--------

                   V3                              α2                                                     V4
 0-PL3.1 PNNNTRKSVR IGPGQTFYAT GEIIGDIRQA YCNISEQTWN DTLQKVGKKL KEQFPNKEIE FAPSSGGDLE ITTHSFNCRG EFFYCNTSKL FNSTYMANST  400
 0-PB3.1 ---------- ---------- ---------- ---------- ---------- ---------- ---------- ---------- ---------- ----------
 5-PB1.1 ---------- ---------- ---------- -----A---- ---------- ---------- ---------- ---------- ---------- ----------
11-PL5.1 ---------- ---------- ---------- ---------- ---------- -----D---K ---------- ---------- ---------- ----------
14-PL4.1 ---------- ---------- -G-------- ---------- ---------- -----D---K ---------- ---------- ---------- ---A------
17-PL3.1 ---------- ---------- -G-------- -----A---- ---------- -----DN--K ---------- ---------- ---------- ----------
20-PB2.1 ---------- ---------- -D-------- H-----K--- ---------- -------T-- -K-------- ---------- ---------- ----------
23-PL5.1 ---------- ---------- -G-------- ---------- ----E----- -------T-- ---------- ---------- ---------- --G-------
26-PL2.1 ---------- ---------- -D-------- H------A-- ----E----- -------T-- ---------- ---------- ---------- ----------
28-PL1.1 ---------- ---------- -D-------- H-----K--- E--------- -------T-- -K-------- ------T--- ---------- --G-------
28-PL3.1 ---------- ---------- -G-------- H-----R--- ----E--R-- -------T-V ---------- ---------- ---------- --....S---
28-PL5.1 ---------- ---------- -D-------- H-----K--- ----E----- -------T-K --S------- ---------- --------Q- --G-------

                                                                  V5
 0-PL3.1 NSTLNGTITL PCRIKQIINL WQKVGRAVYA PPIAGNITCK SNITGLLLTY DGIYANSNNT TLIFRPGGGD MRDNWRSELY KYKVVEIRPL GVAPTKAKRR  500
 0-PB3.1 ---------- ---------- ---------- ---------- ---------H ---------- ---------- ---------- ---------- ----------
 5-PB1.1 ---------- ---------- ---------- ---------- ---------H --T--N---- ---------- ---------- ---------- ----------
11-PL5.1 ---------- ---------- ---------- ---------- ---------H ---DT--.-K ---------- ---------- ---------- ----------
14-PL4.1 ---------- ---------- ---------- ---------- ---------H ---H---.-N -.-------- ---------- ---------- -------R--
17-PL3.1 ---------- ---------- ---------- ---------- ---------H ---SNSN.-- PV-------- ---------- ---------- -----G----
20-PB2.1 ---------- ---------- ---------- ---------- ---------H ---NNTD..- ---------- ---------- ---------- -----E-R--
23-PL5.1 ---------- ---------- ---------- ---------- --------AH --TE--N..E ---------- ---------- ---------- ----------
26-PL2.1 ---------- ---------- ---------- ---------- ---------H ---NSSD..P -I-------- ---------- ---------- -----E-R--
28-PL1.1 ---------- ---------- ---------- ---------- ---------H ---NNTD..- ---------- ---------- ---------- -----E-R--
28-PL3.1 ---------- ---------- --R----M-- ---------- ---------H ---NNTD..- --T------- ---------- ---------- -----E-R--
28-PL5.1 --N------- ---------- ---------- ---------- ---------H ---NSSD..P -IT------- ---------- ---------- -----E-R--

                 FP                                                 HR1
 0-PL3.1 VVEREKRAVG IGAVFLGFLG AAGSTMGAAS ITLTVQARQL LSGIVQQQSN LLRAIEAQQH MLQLTVWGIK QLQTRVLAIE RYLKDQQLLG LWGCSGKLIC  600
 0-PB3.1 ---------- ---------- ---------- ---------- ---------- ---------- ---------- ---------- ---------- ----------
 5-PB1.1 ---------- ---------- ---------- ---------- ---------- ---------- ---------- ---------- ---------- ----------
11-PL5.1 ---------- ---------- ---------- ---------- ---------- ---------- ---------- ---------- ---------- ----------
14-PL4.1 ---------- ---------- ---------- ---------- ---------- ---------- ---------- ---------- ---------- ----------
17-PL3.1 ---------- ---------- ---------- ---------- ---------- ---------- ---------- ---------- ---------- ----------
20-PB2.1 ---------- ---M------ ---------- ---------- ---------- ---------- ---------- ---------- ---------- ----------
23-PL5.1 ---------- ---M------ ---------- ---------- ---------- ---------- ---------- ---------- ---------- ----------
26-PL2.1 ---------- ---M------ ---------- ---------- ---------- ---------- ---------- ---------- ---------- ----------
28-PL1.1 ---------- ---M------ ---------- ---------- ---------- ---------- ---------- ---------- ---------- ----------
28-PL3.1 ---------- ---M------ ---------- ---------- ---------- ---------- ---------- ---------- ---------- ----------
28-PL5.1 ---------- ---------- ---------- ---------- ---------- ---------- ---------- ---------- ---------- ----------

                                             HR2
 0-PL3.1 TTNVPWNYSW SNKTRDEIWD NMTWMQWDRE INNYTDTIYR LLEDSQNQQE KNERDLLALD SWNNLWSWFD ISRWLWYIKI FIMIVGGLIG LRIIFAVLSV  700
 0-PB3.1 ---------- ---------- ---------- ---------- ---------- ---------- ---------- ---------- ---------- ----------
 5-PB1.1 ---------- ---------- ---------- -----N---- ---------- ---------- ---------- ---------- ---------- ---------I
11-PL5.1 ---------- ---------- ---------- -----N---- ---------- ---------- ---------- ---------- ---------- ----------
14-PL4.1 ---------- -----N---- ---------- -----N---- ---------- ---------- ---------- ---------- --T------- ----------
17-PL3.1 -------H-- ---------E ---------- -----N---- ---------- ---------- ---------- ---------- ---------- ----------
20-PB2.1 -------Q-- ---------E ---------- -----N---- ---V------ ---------- ---------- ---------- ---------- ----------
23-PL5.1 ---------- ----T----E ---------- -----N---- ---------- ---------- ---------- ---------- ---------- ----------
26-PL2.1 ---------- ----TN---E ---------- -----G---- ---------- ---------- ---------E ---------- ---------- ----------
28-PL1.1 ---------- ----TN---E ---------- -----N---- ---------- ---------- ---------- ---------- ---------- ----------
28-PL3.1 ---------- --R--N---- ---------- -----N---- ---------- ---------- ---------- ---------- ---------- ----------
28-PL5.1 -------S-- ----TN---- ---------- -----G---- ---------- ---------- ---------N ---------- ---------- ----------

 0-PL3.1 VNRVRQGYSP LSFQTLIPNP RGPDRPGGIE EEGGEQDRDR SVRLVNGFLA IVWDDLRSLC LFSYHQLRDF ILIVARAVEL LGRRGREALK YLGNLVQYWG  800
 0-PB3.1 ---------- ---------- ---------- ---------- ---------- ---------- ---------- ---------- -----W---- ----------
 5-PB1.1 ---------- ---------- ---------- ---------- ---------- ---------- ---------- ---------- -----W---- ---------S
11-PL5.1 ---------- -----P---- ---------- ---------- ---------- ---------- ---------- ---------- -----W---- ----------
14-PL4.1 ---------- ---------- ---------- ---------- ---------- ---------- ---------- ---------- -----W---- ----------
17-PL3.1 ---------- ---------- ---------- ---------- ---------- S--------- ---------- ---------- -----W---- ---S------
20-PB2.1 ---------- ---------- ---------- ---------- ---------- ---------- ---------- ---------- -----W---- ---S------
23-PL5.1 ---------- ---------- ---------- ---------- ---------- ---------- ---------- ---------- -----W---- ----------
26-PL2.1 ---------- ---------- ------R--- ---------- ---------- ---------- ---------- ---------- -----W---- ---S------
28-PL1.1 ---------- ---------- ---------- ---------- ---------- ---------- ---------- ---------- -----W---- ---S------
28-PL3.1 ---------- ---------- ---------- ---------- ---------- ---------- ---------- ---------- -----W---- ---S------
28-PL5.1 ---------- ---------R --L---R--- ---------- ---------- ---------- ---------- ---------- -----W---- ---S------

 0-PL3.1 LELKKSAISL LDTIAITVAE GTDRIIELIQ RIWRAILNIP RRIRQGFEAT LL  852
 0-PB3.1 ---------- ---------- ---------- ------P--- ---------- --
 5-PB1.1 ---------- ---------- ------G--- ---------- ---------- --
11-PL5.1 ---------- ---------- --------T- ---------- ---------A --
14-PL4.1 ---------- ---------- --------T- ---------- ---------- --
17-PL3.1 ---------- ---------- ------G--- ---------- ---------A --
20-PB2.1 ---------- ---------- --------T- ---------- ---------A --
23-PL5.1 ---------- ----V----- --------T- ---------- ---------A --
26-PL2.1 ---------- ---------- --------T- ---------- ---------A --
28-PL1.1 ---------- ---------- --------T- ---------- ---------A --
28-PL3.1 ---------- ---------- ------G-T- ---------- ---------A --
28-PL5.1 ---------- ---------- --------T- ---------- ---------A --
```
